# Search for electroweak production of supersymmetric particles in final states with two or three leptons at $\sqrt{s}=13\hbox {TeV}$ with the ATLAS detector

**DOI:** 10.1140/epjc/s10052-018-6423-7

**Published:** 2018-12-05

**Authors:** M. Aaboud, G. Aad, B. Abbott, O. Abdinov, B. Abeloos, S. H. Abidi, O. S. AbouZeid, N. L. Abraham, H. Abramowicz, H. Abreu, R. Abreu, Y. Abulaiti, B. S. Acharya, S. Adachi, L. Adamczyk, J. Adelman, M. Adersberger, T. Adye, A. A. Affolder, Y. Afik, T. Agatonovic-Jovin, C. Agheorghiesei, J. A. Aguilar-Saavedra, F. Ahmadov, G. Aielli, S. Akatsuka, H. Akerstedt, T. P. A. Åkesson, E. Akilli, A. V. Akimov, G. L. Alberghi, J. Albert, P. Albicocco, M. J. Alconada Verzini, S. Alderweireldt, M. Aleksa, I. N. Aleksandrov, C. Alexa, G. Alexander, T. Alexopoulos, M. Alhroob, B. Ali, G. Alimonti, J. Alison, S. P. Alkire, B. M. M. Allbrooke, B. W. Allen, P. P. Allport, A. Aloisio, A. Alonso, F. Alonso, C. Alpigiani, A. A. Alshehri, M. I. Alstaty, B. Alvarez Gonzalez, D. Álvarez Piqueras, M. G. Alviggi, B. T. Amadio, Y. Amaral Coutinho, C. Amelung, D. Amidei, S. P. Amor Dos Santos, S. Amoroso, G. Amundsen, C. Anastopoulos, L. S. Ancu, N. Andari, T. Andeen, C. F. Anders, J. K. Anders, K. J. Anderson, A. Andreazza, V. Andrei, S. Angelidakis, I. Angelozzi, A. Angerami, A. V. Anisenkov, N. Anjos, A. Annovi, C. Antel, M. Antonelli, A. Antonov, D. J. A. Antrim, F. Anulli, M. Aoki, L. Aperio Bella, G. Arabidze, Y. Arai, J. P. Araque, V. Araujo Ferraz, A. T. H. Arce, R. E. Ardell, F. A. Arduh, J.-F. Arguin, S. Argyropoulos, M. Arik, A. J. Armbruster, L. J. Armitage, O. Arnaez, H. Arnold, M. Arratia, O. Arslan, A. Artamonov, G. Artoni, S. Artz, S. Asai, N. Asbah, A. Ashkenazi, L. Asquith, K. Assamagan, R. Astalos, M. Atkinson, N. B. Atlay, K. Augsten, G. Avolio, B. Axen, M. K. Ayoub, G. Azuelos, A. E. Baas, M. J. Baca, H. Bachacou, K. Bachas, M. Backes, P. Bagnaia, M. Bahmani, H. Bahrasemani, J. T. Baines, M. Bajic, O. K. Baker, E. M. Baldin, P. Balek, F. Balli, W. K. Balunas, E. Banas, A. Bandyopadhyay, S. Banerjee, A. A. E. Bannoura, L. Barak, E. L. Barberio, D. Barberis, M. Barbero, T. Barillari, M.-S. Barisits, J. Barkeloo, T. Barklow, N. Barlow, S. L. Barnes, B. M. Barnett, R. M. Barnett, Z. Barnovska-Blenessy, A. Baroncelli, G. Barone, A. J. Barr, L. Barranco Navarro, F. Barreiro, J. Barreiro Guimarães da Costa, R. Bartoldus, A. E. Barton, P. Bartos, A. Basalaev, A. Bassalat, R. L. Bates, S. J. Batista, J. R. Batley, M. Battaglia, M. Bauce, F. Bauer, H. S. Bawa, J. B. Beacham, M. D. Beattie, T. Beau, P. H. Beauchemin, P. Bechtle, H. C. Beck, H. P. Beck, K. Becker, M. Becker, C. Becot, A. Beddall, A. J. Beddall, V. A. Bednyakov, M. Bedognetti, C. P. Bee, T. A. Beermann, M. Begalli, M. Begel, J. K. Behr, A. S. Bell, G. Bella, L. Bellagamba, A. Bellerive, M. Bellomo, K. Belotskiy, O. Beltramello, N. L. Belyaev, O. Benary, D. Benchekroun, M. Bender, K. Bendtz, N. Benekos, Y. Benhammou, E. Benhar Noccioli, J. Benitez, D. P. Benjamin, M. Benoit, J. R. Bensinger, S. Bentvelsen, L. Beresford, M. Beretta, D. Berge, E. Bergeaas Kuutmann, N. Berger, J. Beringer, S. Berlendis, N. R. Bernard, G. Bernardi, C. Bernius, F. U. Bernlochner, T. Berry, P. Berta, C. Bertella, G. Bertoli, F. Bertolucci, I. A. Bertram, C. Bertsche, D. Bertsche, G. J. Besjes, O. Bessidskaia Bylund, M. Bessner, N. Besson, A. Bethani, S. Bethke, A. J. Bevan, J. Beyer, R. M. Bianchi, O. Biebel, D. Biedermann, R. Bielski, K. Bierwagen, N. V. Biesuz, M. Biglietti, T. R. V. Billoud, H. Bilokon, M. Bindi, A. Bingul, C. Bini, S. Biondi, T. Bisanz, C. Bittrich, D. M. Bjergaard, J. E. Black, K. M. Black, R. E. Blair, T. Blazek, I. Bloch, C. Blocker, A. Blue, W. Blum, U. Blumenschein, Dr. Blunier, G. J. Bobbink, V. S. Bobrovnikov, S. S. Bocchetta, A. Bocci, C. Bock, M. Boehler, D. Boerner, D. Bogavac, A. G. Bogdanchikov, C. Bohm, V. Boisvert, P. Bokan, T. Bold, A. S. Boldyrev, A. E. Bolz, M. Bomben, M. Bona, M. Boonekamp, A. Borisov, G. Borissov, J. Bortfeldt, D. Bortoletto, V. Bortolotto, D. Boscherini, M. Bosman, J. D. Bossio Sola, J. Boudreau, J. Bouffard, E. V. Bouhova-Thacker, D. Boumediene, C. Bourdarios, S. K. Boutle, A. Boveia, J. Boyd, I. R. Boyko, A. J. Bozson, J. Bracinik, A. Brandt, G. Brandt, O. Brandt, U. Bratzler, B. Brau, J. E. Brau, W. D. Breaden Madden, K. Brendlinger, A. J. Brennan, L. Brenner, R. Brenner, S. Bressler, D. L. Briglin, T. M. Bristow, D. Britton, D. Britzger, I. Brock, R. Brock, G. Brooijmans, T. Brooks, W. K. Brooks, J. Brosamer, E. Brost, J. H Broughton, P. A. Bruckman de Renstrom, D. Bruncko, A. Bruni, G. Bruni, L. S. Bruni, S. Bruno, B. H. Brunt, M. Bruschi, N. Bruscino, P. Bryant, L. Bryngemark, T. Buanes, Q. Buat, P. Buchholz, A. G. Buckley, I. A. Budagov, M. K. Bugge, F. Bührer, O. Bulekov, D. Bullock, T. J. Burch, S. Burdin, C. D. Burgard, A. M. Burger, B. Burghgrave, K. Burka, S. Burke, I. Burmeister, J. T. P. Burr, E. Busato, D. Büscher, V. Büscher, P. Bussey, J. M. Butler, C. M. Buttar, J. M. Butterworth, P. Butti, W. Buttinger, A. Buzatu, A. R. Buzykaev, S. Cabrera Urbán, D. Caforio, V. M. M. Cairo, O. Cakir, N. Calace, P. Calafiura, A. Calandri, G. Calderini, P. Calfayan, G. Callea, L. P. Caloba, S. Calvente Lopez, D. Calvet, S. Calvet, T. P. Calvet, R. Camacho Toro, S. Camarda, P. Camarri, D. Cameron, R. Caminal Armadans, C. Camincher, S. Campana, M. Campanelli, A. Camplani, A. Campoverde, V. Canale, M. Cano Bret, J. Cantero, T. Cao, M. D. M. Capeans Garrido, I. Caprini, M. Caprini, M. Capua, R. M. Carbone, R. Cardarelli, F. C. Cardillo, I. Carli, T. Carli, G. Carlino, B. T. Carlson, L. Carminati, R. M. D. Carney, S. Caron, E. Carquin, S. Carrá, G. D. Carrillo-Montoya, D. Casadei, M. P. Casado, M. Casolino, D. W. Casper, R. Castelijn, V. Castillo Gimenez, N. F. Castro, A. Catinaccio, J. R. Catmore, A. Cattai, J. Caudron, V. Cavaliere, E. Cavallaro, D. Cavalli, M. Cavalli-Sforza, V. Cavasinni, E. Celebi, F. Ceradini, L. Cerda Alberich, A. S. Cerqueira, A. Cerri, L. Cerrito, F. Cerutti, A. Cervelli, S. A. Cetin, A. Chafaq, D. Chakraborty, S. K. Chan, W. S. Chan, Y. L. Chan, P. Chang, J. D. Chapman, D. G. Charlton, C. C. Chau, C. A. Chavez Barajas, S. Che, S. Cheatham, A. Chegwidden, S. Chekanov, S. V. Chekulaev, G. A. Chelkov, M. A. Chelstowska, C. Chen, C. H. Chen, H. Chen, J. Chen, S. Chen, S. J. Chen, X. Chen, Y. Chen, H. C. Cheng, H. J. Cheng, A. Cheplakov, E. Cheremushkina, R. Cherkaoui El Moursli, E. Cheu, K. Cheung, L. Chevalier, V. Chiarella, G. Chiarelli, G. Chiodini, A. S. Chisholm, A. Chitan, Y. H. Chiu, M. V. Chizhov, K. Choi, A. R. Chomont, S. Chouridou, Y. S. Chow, V. Christodoulou, M. C. Chu, J. Chudoba, A. J. Chuinard, J. J. Chwastowski, L. Chytka, A. K. Ciftci, D. Cinca, V. Cindro, I. A. Cioară, C. Ciocca, A. Ciocio, F. Cirotto, Z. H. Citron, M. Citterio, M. Ciubancan, A. Clark, B. L. Clark, M. R. Clark, P. J. Clark, R. N. Clarke, C. Clement, Y. Coadou, M. Cobal, A. Coccaro, J. Cochran, L. Colasurdo, B. Cole, A. P. Colijn, J. Collot, T. Colombo, P. Conde Muiño, E. Coniavitis, S. H. Connell, I. A. Connelly, S. Constantinescu, G. Conti, F. Conventi, M. Cooke, A. M. Cooper-Sarkar, F. Cormier, K. J. R. Cormier, M. Corradi, F. Corriveau, A. Cortes-Gonzalez, G. Cortiana, G. Costa, M. J. Costa, D. Costanzo, G. Cottin, G. Cowan, B. E. Cox, K. Cranmer, S. J. Crawley, R. A. Creager, G. Cree, S. Crépé-Renaudin, F. Crescioli, W. A. Cribbs, M. Cristinziani, V. Croft, G. Crosetti, A. Cueto, T. Cuhadar Donszelmann, A. R. Cukierman, J. Cummings, M. Curatolo, J. Cúth, S. Czekierda, P. Czodrowski, M. J. Da Cunha Sargedas De Sousa, C. Da Via, W. Dabrowski, T. Dado, T. Dai, O. Dale, F. Dallaire, C. Dallapiccola, M. Dam, G. D’amen, J. R. Dandoy, M. F. Daneri, N. P. Dang, A. C. Daniells, N. D. Dann, M. Danninger, M. Dano Hoffmann, V. Dao, G. Darbo, S. Darmora, J. Dassoulas, A. Dattagupta, T. Daubney, S. D’Auria, W. Davey, C. David, T. Davidek, D. R. Davis, P. Davison, E. Dawe, I. Dawson, K. De, R. De Asmundis, A. De Benedetti, S. De Castro, S. De Cecco, N. De Groot, P. de Jong, H. De la Torre, F. De Lorenzi, A. De Maria, D. De Pedis, A. De Salvo, U. De Sanctis, A. De Santo, K. De Vasconcelos Corga, J. B. De Vivie De Regie, R. Debbe, C. Debenedetti, D. V. Dedovich, N. Dehghanian, I. Deigaard, M. Del Gaudio, J. Del Peso, D. Delgove, F. Deliot, C. M. Delitzsch, M. Della Pietra, D. Della Volpe, A. Dell’Acqua, L. Dell’Asta, M. Dell’Orso, M. Delmastro, C. Delporte, P. A. Delsart, D. A. DeMarco, S. Demers, M. Demichev, A. Demilly, S. P. Denisov, D. Denysiuk, L. D’Eramo, D. Derendarz, J. E. Derkaoui, F. Derue, P. Dervan, K. Desch, C. Deterre, K. Dette, M. R. Devesa, P. O. Deviveiros, A. Dewhurst, S. Dhaliwal, F. A. Di Bello, A. Di Ciaccio, L. Di Ciaccio, W. K. Di Clemente, C. Di Donato, A. Di Girolamo, B. Di Girolamo, B. Di Micco, R. Di Nardo, K. F. Di Petrillo, A. Di Simone, R. Di Sipio, D. Di Valentino, C. Diaconu, M. Diamond, F. A. Dias, M. A. Diaz, E. B. Diehl, J. Dietrich, S. Díez Cornell, A. Dimitrievska, J. Dingfelder, P. Dita, S. Dita, F. Dittus, F. Djama, T. Djobava, J. I. Djuvsland, M. A. B. Do Vale, D. Dobos, M. Dobre, C. Doglioni, J. Dolejsi, Z. Dolezal, M. Donadelli, S. Donati, P. Dondero, J. Donini, M. D’Onofrio, J. Dopke, A. Doria, M. T. Dova, A. T. Doyle, E. Drechsler, M. Dris, Y. Du, J. Duarte-Campderros, A. Dubreuil, E. Duchovni, G. Duckeck, A. Ducourthial, O. A. Ducu, D. Duda, A. Dudarev, A. C. Dudder, E. M. Duffield, L. Duflot, M. Dührssen, C. Dülsen, M. Dumancic, A. E. Dumitriu, A. K. Duncan, M. Dunford, H. DuranYildiz, M. Düren, A. Durglishvili, D. Duschinger, B. Dutta, D. Duvnjak, M. Dyndal, B. S. Dziedzic, C. Eckardt, K. M. Ecker, R. C. Edgar, T. Eifert, G. Eigen, K. Einsweiler, T. Ekelof, M. El Kacimi, R. El Kosseifi, V. Ellajosyula, M. Ellert, S. Elles, F. Ellinghaus, A. A. Elliot, N. Ellis, J. Elmsheuser, M. Elsing, D. Emeliyanov, Y. Enari, O. C. Endner, J. S. Ennis, J. Erdmann, A. Ereditato, M. Ernst, S. Errede, M. Escalier, C. Escobar, B. Esposito, O. Estrada Pastor, A. I. Etienvre, E. Etzion, H. Evans, A. Ezhilov, M. Ezzi, F. Fabbri, L. Fabbri, V. Fabiani, G. Facini, R. M. Fakhrutdinov, S. Falciano, R. J. Falla, J. Faltova, Y. Fang, M. Fanti, A. Farbin, A. Farilla, C. Farina, E. M. Farina, T. Farooque, S. Farrell, S. M. Farrington, P. Farthouat, F. Fassi, P. Fassnacht, D. Fassouliotis, M. Faucci Giannelli, A. Favareto, W. J. Fawcett, L. Fayard, O. L. Fedin, W. Fedorko, S. Feigl, L. Feligioni, C. Feng, E. J. Feng, H. Feng, M. J. Fenton, A. B. Fenyuk, L. Feremenga, P. Fernandez Martinez, S. Fernandez Perez, J. Ferrando, A. Ferrari, P. Ferrari, R. Ferrari, D. E. Ferreira de Lima, A. Ferrer, D. Ferrere, C. Ferretti, F. Fiedler, M. Filipuzzi, A. Filipčič, F. Filthaut, M. Fincke-Keeler, K. D. Finelli, M. C. N. Fiolhais, L. Fiorini, A. Fischer, C. Fischer, J. Fischer, W. C. Fisher, N. Flaschel, I. Fleck, P. Fleischmann, R. R. M. Fletcher, T. Flick, B. M. Flierl, L. R. Flores Castillo, M. J. Flowerdew, G. T. Forcolin, A. Formica, F. A. Förster, A. C. Forti, A. G. Foster, D. Fournier, H. Fox, S. Fracchia, P. Francavilla, M. Franchini, S. Franchino, D. Francis, L. Franconi, M. Franklin, M. Frate, M. Fraternali, D. Freeborn, S. M. Fressard-Batraneanu, B. Freund, D. Froidevaux, J. A. Frost, C. Fukunaga, T. Fusayasu, J. Fuster, C. Gabaldon, O. Gabizon, A. Gabrielli, A. Gabrielli, G. P. Gach, S. Gadatsch, S. Gadomski, G. Gagliardi, L. G. Gagnon, C. Galea, B. Galhardo, E. J. Gallas, B. J. Gallop, P. Gallus, G. Galster, K. K. Gan, S. Ganguly, Y. Gao, Y. S. Gao, C. García, J. E. García Navarro, J. A. García Pascual, M. Garcia-Sciveres, R. W. Gardner, N. Garelli, V. Garonne, A. Gascon Bravo, K. Gasnikova, C. Gatti, A. Gaudiello, G. Gaudio, I. L. Gavrilenko, C. Gay, G. Gaycken, E. N. Gazis, C. N. P. Gee, J. Geisen, M. Geisen, M. P. Geisler, K. Gellerstedt, C. Gemme, M. H. Genest, C. Geng, S. Gentile, C. Gentsos, S. George, D. Gerbaudo, A. Gershon, G. Gessner, S. Ghasemi, M. Ghneimat, B. Giacobbe, S. Giagu, N. Giangiacomi, P. Giannetti, S. M. Gibson, M. Gignac, M. Gilchriese, D. Gillberg, G. Gilles, D. M. Gingrich, M. P. Giordani, F. M. Giorgi, P. F. Giraud, P. Giromini, G. Giugliarelli, D. Giugni, F. Giuli, C. Giuliani, M. Giulini, B. K. Gjelsten, S. Gkaitatzis, I. Gkialas, E. L. Gkougkousis, P. Gkountoumis, L. K. Gladilin, C. Glasman, J. Glatzer, P. C. F. Glaysher, A. Glazov, M. Goblirsch-Kolb, J. Godlewski, S. Goldfarb, T. Golling, D. Golubkov, A. Gomes, R. Goncalves Gama, J. Goncalves Pinto Firmino Da Costa, R. Gonçalo, G. Gonella, L. Gonella, A. Gongadze, S. González de la Hoz, S. Gonzalez-Sevilla, L. Goossens, P. A. Gorbounov, H. A. Gordon, I. Gorelov, B. Gorini, E. Gorini, A. Gorišek, A. T. Goshaw, C. Gössling, M. I. Gostkin, C. A. Gottardo, C. R. Goudet, D. Goujdami, A. G. Goussiou, N. Govender, E. Gozani, L. Graber, I. Grabowska-Bold, P. O. J. Gradin, J. Gramling, E. Gramstad, S. Grancagnolo, V. Gratchev, P. M. Gravila, C. Gray, H. M. Gray, Z. D. Greenwood, C. Grefe, K. Gregersen, I. M. Gregor, P. Grenier, K. Grevtsov, J. Griffiths, A. A. Grillo, K. Grimm, S. Grinstein, Ph. Gris, J.-F. Grivaz, S. Groh, E. Gross, J. Grosse-Knetter, G. C. Grossi, Z. J. Grout, A. Grummer, L. Guan, W. Guan, J. Guenther, F. Guescini, D. Guest, O. Gueta, B. Gui, E. Guido, T. Guillemin, S. Guindon, U. Gul, C. Gumpert, J. Guo, W. Guo, Y. Guo, R. Gupta, S. Gupta, G. Gustavino, B. J. Gutelman, P. Gutierrez, N. G. Gutierrez Ortiz, C. Gutschow, C. Guyot, M. P. Guzik, C. Gwenlan, C. B. Gwilliam, A. Haas, C. Haber, H. K. Hadavand, N. Haddad, A. Hadef, S. Hageböck, M. Hagihara, H. Hakobyan, M. Haleem, J. Haley, G. Halladjian, G. D. Hallewell, K. Hamacher, P. Hamal, K. Hamano, A. Hamilton, G. N. Hamity, P. G. Hamnett, L. Han, S. Han, K. Hanagaki, K. Hanawa, M. Hance, B. Haney, P. Hanke, J. B. Hansen, J. D. Hansen, M. C. Hansen, P. H. Hansen, K. Hara, A. S. Hard, T. Harenberg, F. Hariri, S. Harkusha, P. F. Harrison, N. M. Hartmann, Y. Hasegawa, A. Hasib, S. Hassani, S. Haug, R. Hauser, L. Hauswald, L. B. Havener, M. Havranek, C. M. Hawkes, R. J. Hawkings, D. Hayakawa, D. Hayden, C. P. Hays, J. M. Hays, H. S. Hayward, S. J. Haywood, S. J. Head, T. Heck, V. Hedberg, L. Heelan, S. Heer, K. K. Heidegger, S. Heim, T. Heim, B. Heinemann, J. J. Heinrich, L. Heinrich, C. Heinz, J. Hejbal, L. Helary, A. Held, S. Hellman, C. Helsens, R. C. W. Henderson, Y. Heng, S. Henkelmann, A. M. Henriques Correia, S. Henrot-Versille, G. H. Herbert, H. Herde, V. Herget, Y. Hernández Jiménez, H. Herr, G. Herten, R. Hertenberger, L. Hervas, T. C. Herwig, G. G. Hesketh, N. P. Hessey, J. W. Hetherly, S. Higashino, E. Higón-Rodriguez, K. Hildebrand, E. Hill, J. C. Hill, K. H. Hiller, S. J. Hillier, M. Hils, I. Hinchliffe, M. Hirose, D. Hirschbuehl, B. Hiti, O. Hladik, X. Hoad, J. Hobbs, N. Hod, M. C. Hodgkinson, P. Hodgson, A. Hoecker, M. R. Hoeferkamp, F. Hoenig, D. Hohn, T. R. Holmes, M. Homann, S. Honda, T. Honda, T. M. Hong, B. H. Hooberman, W. H. Hopkins, Y. Horii, A. J. Horton, J.-Y. Hostachy, A. Hostiuc, S. Hou, A. Hoummada, J. Howarth, J. Hoya, M. Hrabovsky, J. Hrdinka, I. Hristova, J. Hrivnac, A. Hrynevich, T. Hryn’ova, P. J. Hsu, S.-C. Hsu, Q. Hu, S. Hu, Y. Huang, Z. Hubacek, F. Hubaut, F. Huegging, T. B. Huffman, E. W. Hughes, G. Hughes, M. Huhtinen, P. Huo, N. Huseynov, J. Huston, J. Huth, G. Iacobucci, G. Iakovidis, I. Ibragimov, L. Iconomidou-Fayard, Z. Idrissi, P. Iengo, O. Igonkina, T. Iizawa, Y. Ikegami, M. Ikeno, Y. Ilchenko, D. Iliadis, N. Ilic, G. Introzzi, P. Ioannou, M. Iodice, K. Iordanidou, V. Ippolito, M. F. Isacson, N. Ishijima, M. Ishino, M. Ishitsuka, C. Issever, S. Istin, F. Ito, J. M. Iturbe Ponce, R. Iuppa, H. Iwasaki, J. M. Izen, V. Izzo, S. Jabbar, P. Jackson, R. M. Jacobs, V. Jain, K. B. Jakobi, K. Jakobs, S. Jakobsen, T. Jakoubek, D. O. Jamin, D. K. Jana, R. Jansky, J. Janssen, M. Janus, P. A. Janus, G. Jarlskog, N. Javadov, T. Javůrek, M. Javurkova, F. Jeanneau, L. Jeanty, J. Jejelava, A. Jelinskas, P. Jenni, C. Jeske, S. Jézéquel, H. Ji, J. Jia, H. Jiang, Y. Jiang, Z. Jiang, S. Jiggins, J. Jimenez Pena, S. Jin, A. Jinaru, O. Jinnouchi, H. Jivan, P. Johansson, K. A. Johns, C. A. Johnson, W. J. Johnson, K. Jon-And, R. W. L. Jones, S. D. Jones, S. Jones, T. J. Jones, J. Jongmanns, P. M. Jorge, J. Jovicevic, X. Ju, A. Juste Rozas, A. Kaczmarska, M. Kado, H. Kagan, M. Kagan, S. J. Kahn, T. Kaji, E. Kajomovitz, C. W. Kalderon, A. Kaluza, S. Kama, A. Kamenshchikov, N. Kanaya, L. Kanjir, V. A. Kantserov, J. Kanzaki, B. Kaplan, L. S. Kaplan, D. Kar, K. Karakostas, N. Karastathis, M. J. Kareem, E. Karentzos, S. N. Karpov, Z. M. Karpova, K. Karthik, V. Kartvelishvili, A. N. Karyukhin, K. Kasahara, L. Kashif, R. D. Kass, A. Kastanas, Y. Kataoka, C. Kato, A. Katre, J. Katzy, K. Kawade, K. Kawagoe, T. Kawamoto, G. Kawamura, E. F. Kay, V. F. Kazanin, R. Keeler, R. Kehoe, J. S. Keller, E. Kellermann, J. J. Kempster, J Kendrick, H. Keoshkerian, O. Kepka, S. Kersten, B. P. Kerševan, R. A. Keyes, M. Khader, F. Khalil-Zada, A. Khanov, A. G. Kharlamov, T. Kharlamova, A. Khodinov, T. J. Khoo, V. Khovanskiy, E. Khramov, J. Khubua, S. Kido, C. R. Kilby, H. Y. Kim, S. H. Kim, Y. K. Kim, N. Kimura, O. M. Kind, B. T. King, D. Kirchmeier, J. Kirk, A. E. Kiryunin, T. Kishimoto, D. Kisielewska, V. Kitali, O. Kivernyk, E. Kladiva, T. Klapdor-Kleingrothaus, M. H. Klein, M. Klein, U. Klein, K. Kleinknecht, P. Klimek, A. Klimentov, R. Klingenberg, T. Klingl, T. Klioutchnikova, P. Kluit, S. Kluth, E. Kneringer, E. B. F. G. Knoops, A. Knue, A. Kobayashi, D. Kobayashi, T. Kobayashi, M. Kobel, M. Kocian, P. Kodys, T. Koffas, E. Koffeman, M. K. Köhler, N. M. Köhler, T. Koi, M. Kolb, I. Koletsou, A. A. Komar, T. Kondo, N. Kondrashova, K. Köneke, A. C. König, T. Kono, R. Konoplich, N. Konstantinidis, R. Kopeliansky, S. Koperny, A. K. Kopp, K. Korcyl, K. Kordas, A. Korn, A. A. Korol, I. Korolkov, E. V. Korolkova, O. Kortner, S. Kortner, T. Kosek, V. V. Kostyukhin, A. Kotwal, A. Koulouris, A. Kourkoumeli-Charalampidi, C. Kourkoumelis, E. Kourlitis, V. Kouskoura, A. B. Kowalewska, R. Kowalewski, T. Z. Kowalski, C. Kozakai, W. Kozanecki, A. S. Kozhin, V. A. Kramarenko, G. Kramberger, D. Krasnopevtsev, M. W. Krasny, A. Krasznahorkay, D. Krauss, J. A. Kremer, J. Kretzschmar, K. Kreutzfeldt, P. Krieger, K. Krizka, K. Kroeninger, H. Kroha, J. Kroll, J. Kroll, J. Kroseberg, J. Krstic, U. Kruchonak, H. Krüger, N. Krumnack, M. C. Kruse, T. Kubota, H. Kucuk, S. Kuday, J. T. Kuechler, S. Kuehn, A. Kugel, F. Kuger, T. Kuhl, V. Kukhtin, R. Kukla, Y. Kulchitsky, S. Kuleshov, Y. P. Kulinich, M. Kuna, T. Kunigo, A. Kupco, T. Kupfer, O. Kuprash, H. Kurashige, L. L. Kurchaninov, Y. A. Kurochkin, M. G. Kurth, V. Kus, E. S. Kuwertz, M. Kuze, J. Kvita, T. Kwan, D. Kyriazopoulos, A. La Rosa, J. L. La Rosa Navarro, L. La Rotonda, F. La Ruffa, C. Lacasta, F. Lacava, J. Lacey, D. P. J. Lack, H. Lacker, D. Lacour, E. Ladygin, R. Lafaye, B. Laforge, S. Lai, S. Lammers, W. Lampl, E. Lançon, U. Landgraf, M. P. J. Landon, M. C. Lanfermann, V. S. Lang, J. C. Lange, R. J. Langenberg, A. J. Lankford, F. Lanni, K. Lantzsch, A. Lanza, A. Lapertosa, S. Laplace, J. F. Laporte, T. Lari, F. Lasagni Manghi, M. Lassnig, T. S. Lau, P. Laurelli, W. Lavrijsen, A. T. Law, P. Laycock, T. Lazovich, M. Lazzaroni, B. Le, O. Le Dortz, E. Le Guirriec, E. P. Le Quilleuc, M. LeBlanc, T. LeCompte, F. Ledroit-Guillon, C. A. Lee, G. R. Lee, L. Lee, S. C. Lee, B. Lefebvre, G. Lefebvre, M. Lefebvre, F. Legger, C. Leggett, G. Lehmann Miotto, X. Lei, W. A. Leight, M. A. L. Leite, R. Leitner, D. Lellouch, B. Lemmer, K. J. C. Leney, T. Lenz, B. Lenzi, R. Leone, S. Leone, C. Leonidopoulos, G. Lerner, C. Leroy, A. A. J. Lesage, C. G. Lester, M. Levchenko, J. Levêque, D. Levin, L. J. Levinson, M. Levy, D. Lewis, B. Li, C.-Q. Li, H. Li, L. Li, Q. Li, Q. Y. Li, S. Li, X. Li, Y. Li, Z. Liang, B. Liberti, A. Liblong, K. Lie, J. Liebal, W. Liebig, A. Limosani, S. C. Lin, T. H. Lin, R. A. Linck, B. E. Lindquist, A. L. Lionti, E. Lipeles, A. Lipniacka, M. Lisovyi, T. M. Liss, A. Lister, A. M. Litke, B. Liu, H. B. Liu, H. Liu, J. B. Liu, J. K. K. Liu, J. Liu, K. Liu, L. Liu, M. Liu, Y. L. Liu, Y. W. Liu, M. Livan, A. Lleres, J. Llorente Merino, S. L. Lloyd, C. Y. Lo, F. Lo Sterzo, E. M. Lobodzinska, P. Loch, F. K. Loebinger, K. M. Loew, A. Loginov, T. Lohse, K. Lohwasser, M. Lokajicek, B. A. Long, J. D. Long, R. E. Long, L. Longo, K. A. Looper, J. A. Lopez, D. Lopez Mateos, I. Lopez Paz, A. Lopez Solis, J. Lorenz, N. Lorenzo Martinez, M. Losada, P. J. Lösel, A. Lösle, X. Lou, A. Lounis, J. Love, P. A. Love, H. Lu, N. Lu, Y. J. Lu, H. J. Lubatti, C. Luci, A. Lucotte, C. Luedtke, F. Luehring, W. Lukas, L. Luminari, O. Lundberg, B. Lund-Jensen, M. S. Lutz, P. M. Luzi, D. Lynn, R. Lysak, E. Lytken, F. Lyu, V. Lyubushkin, H. Ma, L. L. Ma, Y. Ma, G. Maccarrone, A. Macchiolo, C. M. Macdonald, J. Machado Miguens, D. Madaffari, R. Madar, W. F. Mader, A. Madsen, J. Maeda, S. Maeland, T. Maeno, A. S. Maevskiy, V. Magerl, J. Mahlstedt, C. Maiani, C. Maidantchik, A. A. Maier, T. Maier, A. Maio, O. Majersky, S. Majewski, Y. Makida, N. Makovec, B. Malaescu, Pa. Malecki, V. P. Maleev, F. Malek, U. Mallik, D. Malon, C. Malone, S. Maltezos, S. Malyukov, J. Mamuzic, G. Mancini, I. Mandić, J. Maneira, L. Manhaes de Andrade Filho, J. Manjarres Ramos, K. H. Mankinen, A. Mann, A. Manousos, B. Mansoulie, J. D. Mansour, R. Mantifel, M. Mantoani, S. Manzoni, L. Mapelli, G. Marceca, L. March, L. Marchese, G. Marchiori, M. Marcisovsky, C. A. Marin Tobon, M. Marjanovic, D. E. Marley, F. Marroquim, S. P. Marsden, Z. Marshall, M. U. F Martensson, S. Marti-Garcia, C. B. Martin, T. A. Martin, V. J. Martin, B. Martin dit Latour, M. Martinez, V. I. Martinez Outschoorn, S. Martin-Haugh, V. S. Martoiu, A. C. Martyniuk, A. Marzin, L. Masetti, T. Mashimo, R. Mashinistov, J. Masik, A. L. Maslennikov, L. Massa, P. Mastrandrea, A. Mastroberardino, T. Masubuchi, P. Mättig, J. Maurer, B. Maček, S. J. Maxfield, D. A. Maximov, R. Mazini, I. Maznas, S. M. Mazza, N. C. Mc Fadden, G. Mc Goldrick, S. P. Mc Kee, A. McCarn, R. L. McCarthy, T. G. McCarthy, L. I. McClymont, E. F. McDonald, J. A. Mcfayden, G. Mchedlidze, S. J. McMahon, P. C. McNamara, C. J. McNicol, R. A. McPherson, S. Meehan, T. M. Megy, S. Mehlhase, A. Mehta, T. Meideck, B. Meirose, D. Melini, B. R. Mellado Garcia, J. D. Mellenthin, M. Melo, F. Meloni, A. Melzer, S. B. Menary, L. Meng, X. T. Meng, A. Mengarelli, S. Menke, E. Meoni, S. Mergelmeyer, C. Merlassino, P. Mermod, L. Merola, C. Meroni, F. S. Merritt, A. Messina, J. Metcalfe, A. S. Mete, C. Meyer, J. Meyer, J.-P. Meyer, H. Meyer Zu Theenhausen, F. Miano, R. P. Middleton, S. Miglioranzi, L. Mijović, G. Mikenberg, M. Mikestikova, M. Mikuž, M. Milesi, A. Milic, D. A. Millar, D. W. Miller, C. Mills, A. Milov, D. A. Milstead, A. A. Minaenko, Y. Minami, I. A. Minashvili, A. I. Mincer, B. Mindur, M. Mineev, Y. Minegishi, Y. Ming, L. M. Mir, K. P. Mistry, T. Mitani, J. Mitrevski, V. A. Mitsou, A. Miucci, P. S. Miyagawa, A. Mizukami, J. U. Mjörnmark, T. Mkrtchyan, M. Mlynarikova, T. Moa, K. Mochizuki, P. Mogg, S. Mohapatra, S. Molander, R. Moles-Valls, M. C. Mondragon, K. Mönig, J. Monk, E. Monnier, A. Montalbano, J. Montejo Berlingen, F. Monticelli, S. Monzani, R. W. Moore, N. Morange, D. Moreno, M. Moreno Llácer, P. Morettini, S. Morgenstern, D. Mori, T. Mori, M. Morii, M. Morinaga, V. Morisbak, A. K. Morley, G. Mornacchi, J. D. Morris, L. Morvaj, P. Moschovakos, M. Mosidze, H. J. Moss, J. Moss, K. Motohashi, R. Mount, E. Mountricha, E. J. W. Moyse, S. Muanza, F. Mueller, J. Mueller, R. S. P. Mueller, D. Muenstermann, P. Mullen, G. A. Mullier, F. J. Munoz Sanchez, W. J. Murray, H. Musheghyan, M. Muškinja, A. G. Myagkov, M. Myska, B. P. Nachman, O. Nackenhorst, K. Nagai, R. Nagai, K. Nagano, Y. Nagasaka, K. Nagata, M. Nagel, E. Nagy, A. M. Nairz, Y. Nakahama, K. Nakamura, T. Nakamura, I. Nakano, R. F. Naranjo Garcia, R. Narayan, D. I. Narrias Villar, I. Naryshkin, T. Naumann, G. Navarro, R. Nayyar, H. A. Neal, P. Y. Nechaeva, T. J. Neep, A. Negri, M. Negrini, S. Nektarijevic, C. Nellist, A. Nelson, M. E. Nelson, S. Nemecek, P. Nemethy, M. Nessi, M. S. Neubauer, M. Neumann, P. R. Newman, T. Y. Ng, T. Nguyen Manh, R. B. Nickerson, R. Nicolaidou, J. Nielsen, V. Nikolaenko, I. Nikolic-Audit, K. Nikolopoulos, J. K. Nilsen, P. Nilsson, Y. Ninomiya, A. Nisati, N. Nishu, R. Nisius, I. Nitsche, T. Nitta, T. Nobe, Y. Noguchi, M. Nomachi, I. Nomidis, M. A. Nomura, T. Nooney, M. Nordberg, N. Norjoharuddeen, O. Novgorodova, M. Nozaki, L. Nozka, K. Ntekas, E. Nurse, F. Nuti, F. G. Oakham, H. Oberlack, T. Obermann, J. Ocariz, A. Ochi, I. Ochoa, J. P. Ochoa-Ricoux, K. O’Connor, S. Oda, S. Odaka, A. Oh, S. H. Oh, C. C. Ohm, H. Ohman, H. Oide, H. Okawa, Y. Okumura, T. Okuyama, A. Olariu, L. F. Oleiro Seabra, S. A. Olivares Pino, D. Oliveira Damazio, A. Olszewski, J. Olszowska, D. C. O’Neil, A. Onofre, K. Onogi, P. U. E. Onyisi, H. Oppen, M. J. Oreglia, Y. Oren, D. Orestano, N. Orlando, A. A. O’Rourke, R. S. Orr, B. Osculati, V. O’Shea, R. Ospanov, G. Otero y Garzon, H. Otono, M. Ouchrif, F. Ould-Saada, A. Ouraou, K. P. Oussoren, Q. Ouyang, M. Owen, R. E. Owen, V. E. Ozcan, N. Ozturk, H. A. Pacey, K. Pachal, A. Pacheco Pages, L. Pacheco Rodriguez, C. Padilla Aranda, S. Pagan Griso, M. Paganini, F. Paige, G. Palacino, S. Palazzo, S. Palestini, M. Palka, D. Pallin, E. St. Panagiotopoulou, I. Panagoulias, C. E. Pandini, J. G. Panduro Vazquez, P. Pani, S. Panitkin, D. Pantea, L. Paolozzi, T. D. Papadopoulou, K. Papageorgiou, A. Paramonov, D. Paredes Hernandez, A. J. Parker, K. A. Parker, M. A. Parker, F. Parodi, J. A. Parsons, U. Parzefall, V. R. Pascuzzi, J. M. P. Pasner, E. Pasqualucci, S. Passaggio, F. Pastore, S. Pataraia, J. R. Pater, T. Pauly, B. Pearson, S. Pedraza Lopez, R. Pedro, S. V. Peleganchuk, O. Penc, C. Peng, H. Peng, J. Penwell, B. S. Peralva, M. M. Perego, D. V. Perepelitsa, F. Peri, L. Perini, H. Pernegger, S. Perrella, R. Peschke, V. D. Peshekhonov, K. Peters, R. F. Y. Peters, B. A. Petersen, T. C. Petersen, E. Petit, A. Petridis, C. Petridou, P. Petroff, E. Petrolo, M. Petrov, F. Petrucci, N. E. Pettersson, A. Peyaud, R. Pezoa, F. H. Phillips, P. W. Phillips, G. Piacquadio, E. Pianori, A. Picazio, E. Piccaro, M. A. Pickering, R. Piegaia, J. E. Pilcher, A. D. Pilkington, A. W. J. Pin, M. Pinamonti, J. L. Pinfold, H. Pirumov, M. Pitt, L. Plazak, M.-A. Pleier, V. Pleskot, E. Plotnikova, D. Pluth, P. Podberezko, R. Poettgen, R. Poggi, L. Poggioli, I. Pogrebnyak, D. Pohl, G. Polesello, A. Poley, A. Policicchio, R. Polifka, A. Polini, C. S. Pollard, V. Polychronakos, K. Pommès, D. Ponomarenko, L. Pontecorvo, G. A. Popeneciu, D. M. Portillo Quintero, S. Pospisil, K. Potamianos, I. N. Potrap, C. J. Potter, H. Potti, T. Poulsen, J. Poveda, M. E. Pozo Astigarraga, P. Pralavorio, A. Pranko, S. Prell, D. Price, M. Primavera, S. Prince, N. Proklova, K. Prokofiev, F. Prokoshin, S. Protopopescu, J. Proudfoot, M. Przybycien, A. Puri, P. Puzo, J. Qian, G. Qin, Y. Qin, A. Quadt, M. Queitsch-Maitland, D. Quilty, S. Raddum, V. Radeka, V. Radescu, S. K. Radhakrishnan, P. Radloff, P. Rados, F. Ragusa, G. Rahal, J. A. Raine, S. Rajagopalan, C. Rangel-Smith, T. Rashid, S. Raspopov, M. G. Ratti, D. M. Rauch, F. Rauscher, S. Rave, I. Ravinovich, J. H. Rawling, M. Raymond, A. L. Read, N. P. Readioff, M. Reale, D. M. Rebuzzi, A. Redelbach, G. Redlinger, R. Reece, R. G. Reed, K. Reeves, L. Rehnisch, J. Reichert, A. Reiss, C. Rembser, H. Ren, M. Rescigno, S. Resconi, E. D. Resseguie, S. Rettie, E. Reynolds, O. L. Rezanova, P. Reznicek, R. Rezvani, R. Richter, S. Richter, E. Richter-Was, O. Ricken, M. Ridel, P. Rieck, C. J. Riegel, J. Rieger, O. Rifki, M. Rijssenbeek, A. Rimoldi, M. Rimoldi, L. Rinaldi, G. Ripellino, B. Ristić, E. Ritsch, I. Riu, F. Rizatdinova, E. Rizvi, C. Rizzi, R. T. Roberts, S. H. Robertson, A. Robichaud-Veronneau, D. Robinson, J. E. M. Robinson, A. Robson, E. Rocco, C. Roda, Y. Rodina, S. Rodriguez Bosca, A. Rodriguez Perez, D. Rodriguez Rodriguez, S. Roe, C. S. Rogan, O. Røhne, J. Roloff, A. Romaniouk, M. Romano, S. M. Romano Saez, E. Romero Adam, N. Rompotis, M. Ronzani, L. Roos, S. Rosati, K. Rosbach, P. Rose, N.-A. Rosien, E. Rossi, L. P. Rossi, J. H. N. Rosten, R. Rosten, M. Rotaru, J. Rothberg, D. Rousseau, A. Rozanov, Y. Rozen, X. Ruan, F. Rubbo, F. Rühr, A. Ruiz-Martinez, Z. Rurikova, N. A. Rusakovich, H. L. Russell, J. P. Rutherfoord, N. Ruthmann, Y. F. Ryabov, M. Rybar, G. Rybkin, S. Ryu, A. Ryzhov, G. F. Rzehorz, A. F. Saavedra, G. Sabato, S. Sacerdoti, H. F.-W. Sadrozinski, R. Sadykov, F. Safai Tehrani, P. Saha, M. Sahinsoy, M. Saimpert, M. Saito, T. Saito, H. Sakamoto, Y. Sakurai, G. Salamanna, J. E. Salazar Loyola, D. Salek, P. H. Sales De Bruin, D. Salihagic, A. Salnikov, J. Salt, D. Salvatore, F. Salvatore, A. Salvucci, A. Salzburger, D. Sammel, D. Sampsonidis, D. Sampsonidou, J. Sánchez, V. Sanchez Martinez, A. Sanchez Pineda, H. Sandaker, R. L. Sandbach, C. O. Sander, M. Sandhoff, C. Sandoval, D. P. C. Sankey, M. Sannino, Y. Sano, A. Sansoni, C. Santoni, H. Santos, I. Santoyo Castillo, A. Sapronov, J. G. Saraiva, B. Sarrazin, O. Sasaki, K. Sato, E. Sauvan, G. Savage, P. Savard, N. Savic, C. Sawyer, L. Sawyer, J. Saxon, C. Sbarra, A. Sbrizzi, T. Scanlon, D. A. Scannicchio, J. Schaarschmidt, P. Schacht, B. M. Schachtner, D. Schaefer, L. Schaefer, R. Schaefer, J. Schaeffer, S. Schaepe, S. Schaetzel, U. Schäfer, A. C. Schaffer, D. Schaile, R. D. Schamberger, V. A. Schegelsky, D. Scheirich, M. Schernau, C. Schiavi, S. Schier, L. K. Schildgen, C. Schillo, M. Schioppa, S. Schlenker, K. R. Schmidt-Sommerfeld, K. Schmieden, C. Schmitt, S. Schmitt, S. Schmitz, U. Schnoor, L. Schoeffel, A. Schoening, B. D. Schoenrock, E. Schopf, M. Schott, J. F. P. Schouwenberg, J. Schovancova, S. Schramm, N. Schuh, A. Schulte, M. J. Schultens, H.-C. Schultz-Coulon, H. Schulz, M. Schumacher, B. A. Schumm, Ph. Schune, A. Schwartzman, T. A. Schwarz, H. Schweiger, Ph. Schwemling, R. Schwienhorst, A. Sciandra, G. Sciolla, M. Scornajenghi, F. Scuri, F. Scutti, J. Searcy, P. Seema, S. C. Seidel, A. Seiden, J. M. Seixas, G. Sekhniaidze, K. Sekhon, S. J. Sekula, N. Semprini-Cesari, S. Senkin, C. Serfon, L. Serin, L. Serkin, M. Sessa, R. Seuster, H. Severini, T. Šfiligoj, F. Sforza, A. Sfyrla, E. Shabalina, N. W. Shaikh, L. Y. Shan, R. Shang, J. T. Shank, M. Shapiro, A. S. Sharma, P. B. Shatalov, K. Shaw, S. M. Shaw, A. Shcherbakova, C. Y. Shehu, Y. Shen, N. Sherafati, P. Sherwood, L. Shi, S. Shimizu, C. O. Shimmin, M. Shimojima, I. P. J. Shipsey, S. Shirabe, M. Shiyakova, J. Shlomi, A. Shmeleva, D. Shoaleh Saadi, M. J. Shochet, S. Shojaii, D. R. Shope, S. Shrestha, E. Shulga, M. A. Shupe, P. Sicho, A. M. Sickles, P. E. Sidebo, E. Sideras Haddad, O. Sidiropoulou, A. Sidoti, F. Siegert, Dj. Sijacki, J. Silva, S. B. Silverstein, V. Simak, L. Simic, S. Simion, E. Simioni, B. Simmons, M. Simon, P. Sinervo, N. B. Sinev, M. Sioli, G. Siragusa, I. Siral, S. Yu. Sivoklokov, J. Sjölin, M. B. Skinner, P. Skubic, M. Slater, T. Slavicek, M. Slawinska, K. Sliwa, R. Slovak, V. Smakhtin, B. H. Smart, J. Smiesko, N. Smirnov, S. Yu. Smirnov, Y. Smirnov, L. N. Smirnova, O. Smirnova, J. W. Smith, M. N. K. Smith, R. W. Smith, M. Smizanska, K. Smolek, A. A. Snesarev, I. M. Snyder, S. Snyder, R. Sobie, F. Socher, A. Soffer, A. Søgaard, D. A. Soh, G. Sokhrannyi, C. A. Solans Sanchez, M. Solar, E. Yu. Soldatov, U. Soldevila, A. A. Solodkov, A. Soloshenko, O. V. Solovyanov, V. Solovyev, P. Sommer, H. Son, A. Sopczak, D. Sosa, C. L. Sotiropoulou, R. Soualah, A. M. Soukharev, D. South, B. C. Sowden, S. Spagnolo, M. Spalla, M. Spangenberg, F. Spanò, D. Sperlich, F. Spettel, T. M. Spieker, R. Spighi, G. Spigo, L. A. Spiller, M. Spousta, R. D. St. Denis, A. Stabile, R. Stamen, S. Stamm, E. Stanecka, R. W. Stanek, C. Stanescu, M. M. Stanitzki, B. Stapf, S. Stapnes, E. A. Starchenko, G. H. Stark, J. Stark, S. H. Stark, P. Staroba, P. Starovoitov, S. Stärz, R. Staszewski, M. Stegler, P. Steinberg, B. Stelzer, H. J. Stelzer, O. Stelzer-Chilton, H. Stenzel, G. A. Stewart, M. C. Stockton, M. Stoebe, G. Stoicea, P. Stolte, S. Stonjek, A. R. Stradling, A. Straessner, M. E. Stramaglia, J. Strandberg, S. Strandberg, M. Strauss, P. Strizenec, R. Ströhmer, D. M. Strom, R. Stroynowski, A. Strubig, S. A. Stucci, B. Stugu, N. A. Styles, D. Su, J. Su, S. Suchek, Y. Sugaya, M. Suk, V. V. Sulin, D. M. S. Sultan, S. Sultansoy, T. Sumida, S. Sun, X. Sun, K. Suruliz, C. J. E. Suster, M. R. Sutton, S. Suzuki, M. Svatos, M. Swiatlowski, S. P. Swift, I. Sykora, T. Sykora, D. Ta, K. Tackmann, J. Taenzer, A. Taffard, R. Tafirout, E. Tahirovic, N. Taiblum, H. Takai, R. Takashima, E. H. Takasugi, T. Takeshita, Y. Takubo, M. Talby, A. A. Talyshev, J. Tanaka, M. Tanaka, R. Tanaka, S. Tanaka, R. Tanioka, B. B. Tannenwald, S. Tapia Araya, S. Tapprogge, S. Tarem, G. F. Tartarelli, P. Tas, M. Tasevsky, T. Tashiro, E. Tassi, A. Tavares Delgado, Y. Tayalati, A. C. Taylor, A. J. Taylor, G. N. Taylor, P. T. E. Taylor, W. Taylor, P. Teixeira-Dias, D. Temple, H. Ten Kate, P. K. Teng, J. J. Teoh, F. Tepel, S. Terada, K. Terashi, J. Terron, S. Terzo, M. Testa, R. J. Teuscher, T. Theveneaux-Pelzer, F. Thiele, J. P. Thomas, J. Thomas-Wilsker, A. S. Thompson, P. D. Thompson, L. A. Thomsen, E. Thomson, M. J. Tibbetts, R. E. Ticse Torres, V. O. Tikhomirov, Yu. A. Tikhonov, S. Timoshenko, P. Tipton, S. Tisserant, K. Todome, S. Todorova-Nova, S. Todt, J. Tojo, S. Tokár, K. Tokushuku, E. Tolley, L. Tomlinson, M. Tomoto, L. Tompkins, K. Toms, B. Tong, P. Tornambe, E. Torrence, H. Torres, E. Torró Pastor, J. Toth, F. Touchard, D. R. Tovey, C. J. Treado, T. Trefzger, F. Tresoldi, A. Tricoli, I. M. Trigger, S. Trincaz-Duvoid, M. F. Tripiana, W. Trischuk, B. Trocmé, A. Trofymov, C. Troncon, M. Trottier-McDonald, M. Trovatelli, L. Truong, M. Trzebinski, A. Trzupek, K. W. Tsang, J. C.-L. Tseng, P. V. Tsiareshka, G. Tsipolitis, N. Tsirintanis, S. Tsiskaridze, V. Tsiskaridze, E. G. Tskhadadze, K. M. Tsui, I. I. Tsukerman, V. Tsulaia, S. Tsuno, D. Tsybychev, Y. Tu, A. Tudorache, V. Tudorache, T. T. Tulbure, A. N. Tuna, S. A. Tupputi, S. Turchikhin, D. Turgeman, I. Turk Cakir, R. Turra, P. M. Tuts, G. Ucchielli, I. Ueda, M. Ughetto, F. Ukegawa, G. Unal, A. Undrus, G. Unel, F. C. Ungaro, Y. Unno, C. Unverdorben, J. Urban, P. Urquijo, P. Urrejola, G. Usai, J. Usui, L. Vacavant, V. Vacek, B. Vachon, K. O. H. Vadla, A. Vaidya, C. Valderanis, E. Valdes Santurio, M. Valente, S. Valentinetti, A. Valero, L. Valéry, S. Valkar, A. Vallier, J. A. Valls Ferrer, W. Van Den Wollenberg, H. Van der Graaf, P. Van Gemmeren, J. Van Nieuwkoop, I. Van Vulpen, M. C. van Woerden, M. Vanadia, W. Vandelli, A. Vaniachine, P. Vankov, G. Vardanyan, R. Vari, E. W. Varnes, C. Varni, T. Varol, D. Varouchas, A. Vartapetian, K. E. Varvell, G. A. Vasquez, J. G. Vasquez, F. Vazeille, D. Vazquez Furelos, T. Vazquez Schroeder, J. Veatch, V. Veeraraghavan, L. M. Veloce, F. Veloso, S. Veneziano, A. Ventura, M. Venturi, N. Venturi, A. Venturini, V. Vercesi, M. Verducci, W. Verkerke, A. T. Vermeulen, J. C. Vermeulen, M. C. Vetterli, N. Viaux Maira, O. Viazlo, I. Vichou, T. Vickey, O. E. Vickey Boeriu, G. H. A. Viehhauser, S. Viel, L. Vigani, M. Villa, M. Villaplana Perez, E. Vilucchi, M. G. Vincter, V. B. Vinogradov, A. Vishwakarma, C. Vittori, I. Vivarelli, S. Vlachos, M. Vogel, P. Vokac, G. Volpi, H. von der Schmitt, E. Von Toerne, V. Vorobel, K. Vorobev, M. Vos, R. Voss, J. H. Vossebeld, N. Vranjes, M. Vranjes Milosavljevic, V. Vrba, M. Vreeswijk, R. Vuillermet, I. Vukotic, P. Wagner, W. Wagner, J. Wagner-Kuhr, H. Wahlberg, S. Wahrmund, J. Walder, R. Walker, W. Walkowiak, V. Wallangen, C. Wang, C. Wang, F. Wang, H. Wang, H. Wang, J. Wang, J. Wang, Q. Wang, R. Wang, S. M. Wang, T. Wang, W. Wang, W. X. Wang, Z. Wang, C. Wanotayaroj, A. Warburton, C. P. Ward, D. R. Wardrope, A. Washbrook, P. M. Watkins, A. T. Watson, M. F. Watson, G. Watts, S. Watts, B. M. Waugh, A. F. Webb, S. Webb, M. S. Weber, S. A. Weber, S. W. Weber, J. S. Webster, A. R. Weidberg, B. Weinert, J. Weingarten, M. Weirich, C. Weiser, H. Weits, P. S. Wells, T. Wenaus, T. Wengler, S. Wenig, N. Wermes, M. D. Werner, P. Werner, M. Wessels, T. D. Weston, K. Whalen, N. L. Whallon, A. M. Wharton, A. S. White, A. White, M. J. White, R. White, D. Whiteson, B. W. Whitmore, F. J. Wickens, W. Wiedenmann, M. Wielers, C. Wiglesworth, L. A. M. Wiik-Fuchs, A. Wildauer, F. Wilk, H. G. Wilkens, H. H. Williams, S. Williams, C. Willis, S. Willocq, J. A. Wilson, I. Wingerter-Seez, E. Winkels, F. Winklmeier, O. J. Winston, B. T. Winter, M. Wittgen, M. Wobisch, T. M. H. Wolf, R. Wolff, M. W. Wolter, H. Wolters, V. W. S. Wong, S. D. Worm, B. K. Wosiek, J. Wotschack, K. W. Woźniak, M. Wu, S. L. Wu, X. Wu, Y. Wu, T. R. Wyatt, B. M. Wynne, S. Xella, Z. Xi, L. Xia, D. Xu, L. Xu, T. Xu, B. Yabsley, S. Yacoob, D. Yamaguchi, Y. Yamaguchi, A. Yamamoto, S. Yamamoto, T. Yamanaka, F. Yamane, M. Yamatani, Y. Yamazaki, Z. Yan, H. J. Yang, H. T. Yang, Y. Yang, Z. Yang, W.-M. Yao, Y. C. Yap, Y. Yasu, E. Yatsenko, K. H. Yau Wong, J. Ye, S. Ye, I. Yeletskikh, E. Yigitbasi, E. Yildirim, K. Yorita, K. Yoshihara, C. J. S. Young, C. Young, J. Yu, J. Yu, S. P. Y. Yuen, I. Yusuff, B. Zabinski, G. Zacharis, R. Zaidan, A. M. Zaitsev, N. Zakharchuk, J. Zalieckas, A. Zaman, S. Zambito, D. Zanzi, C. Zeitnitz, G. Zemaityte, A. Zemla, J. C. Zeng, Q. Zeng, O. Zenin, T. Ženiš, D. Zerwas, D. Zhang, F. Zhang, G. Zhang, H. Zhang, J. Zhang, L. Zhang, L. Zhang, M. Zhang, P. Zhang, R. Zhang, R. Zhang, X. Zhang, Y. Zhang, Z. Zhang, X. Zhao, Y. Zhao, Z. Zhao, A. Zhemchugov, B. Zhou, C. Zhou, L. Zhou, M. S. Zhou, M. Zhou, N. Zhou, C. G. Zhu, H. Zhu, J. Zhu, Y. Zhu, X. Zhuang, K. Zhukov, A. Zibell, D. Zieminska, N. I. Zimine, C. Zimmermann, S. Zimmermann, Z. Zinonos, M. Zinser, M. Ziolkowski, L. Živković, G. Zobernig, A. Zoccoli, R. Zou, M. Zur Nedden, L. Zwalinski, ATLAS Collaboration

**Affiliations:** 1 Department of Physics, University of Adelaide, Adelaide, Australia; 2 Physics Department, SUNY Albany, Albany, NY USA; 3 Department of Physics, University of Alberta, Edmonton, AB Canada; 4 Department of Physics, Ankara University, Ankara, Turkey; 5 Istanbul Aydin University, Istanbul, Turkey; 6 Division of Physics, TOBB University of Economics and Technology, Ankara, Turkey; 7 LAPP, Université Grenoble Alpes, Université Savoie Mont Blanc, CNRS/IN2P3, Annecy, France; 8 High Energy Physics Division, Argonne National Laboratory, Argonne, IL USA; 9 Department of Physics, University of Arizona, Tucson, AZ USA; 10 Department of Physics, University of Texas at Arlington, Arlington, TX USA; 11 Physics Department, National and Kapodistrian University of Athens, Athens, Greece; 12 Physics Department, National Technical University of Athens, Zografou, Greece; 13 Department of Physics, University of Texas at Austin, Austin, TX USA; 14 Faculty of Engineering and Natural Sciences, Bahcesehir University, Istanbul, Turkey; 15 Faculty of Engineering and Natural Sciences, Istanbul Bilgi University, Istanbul, Turkey; 16 Department of Physics, Bogazici University, Istanbul, Turkey; 17 Department of Physics Engineering, Gaziantep University, Gaziantep, Turkey; 18 Institute of Physics, Azerbaijan Academy of Sciences, Baku, Azerbaijan; 19 Institut de Física d’Altes Energies (IFAE), Barcelona Institute of Science and Technology, Barcelona, Spain; 20 Institute of High Energy Physics, Chinese Academy of Sciences, Beijing, China; 21 Physics Department, Tsinghua University, Beijing, China; 22 Department of Physics, Nanjing University, Nanjing, China; 23 University of Chinese Academy of Science (UCAS), Beijing, China; 24 Institute of Physics, University of Belgrade, Belgrade, Serbia; 25 Department for Physics and Technology, University of Bergen, Bergen, Norway; 26 Physics Division, Lawrence Berkeley National Laboratory and University of California, Berkeley, CA USA; 27 Institut für Physik, Humboldt Universität zu Berlin, Berlin, Germany; 28 Albert Einstein Center for Fundamental Physics and Laboratory for High Energy Physics, University of Bern, Bern, Switzerland; 29 School of Physics and Astronomy, University of Birmingham, Birmingham, UK; 30 Centro de Investigaciónes, Universidad Antonio Nariño, Bogotá, Colombia; 31 Dipartimento di Fisica e Astronomia, Università di Bologna, Bologna, Italy; 32 INFN Sezione di Bologna, Bologna, Italy; 33 Physikalisches Institut, Universität Bonn, Bonn, Germany; 34 Department of Physics, Boston University, Boston, MA USA; 35 Department of Physics, Brandeis University, Waltham, MA USA; 36 Transilvania University of Brasov, Brasov, Romania; 37 Horia Hulubei National Institute of Physics and Nuclear Engineering, Bucharest, Romania; 38 Department of Physics, Alexandru Ioan Cuza University of Iasi, Iasi, Romania; 39 Physics Department, National Institute for Research and Development of Isotopic and Molecular Technologies, Cluj-Napoca, Romania; 40 University Politehnica Bucharest, Bucharest, Romania; 41 West University in Timisoara, Timisoara, Romania; 42 Faculty of Mathematics, Physics and Informatics, Comenius University, Bratislava, Slovak Republic; 43 Department of Subnuclear Physics, Institute of Experimental Physics of the Slovak Academy of Sciences, Kosice, Slovak Republic; 44 Physics Department, Brookhaven National Laboratory, Upton, NY USA; 45 Departamento de Física, Universidad de Buenos Aires, Buenos Aires, Argentina; 46 Cavendish Laboratory, University of Cambridge, Cambridge, UK; 47 Department of Physics, University of Cape Town, Cape Town, South Africa; 48 Department of Mechanical Engineering Science, University of Johannesburg, Johannesburg, South Africa; 49 School of Physics, University of the Witwatersrand, Johannesburg, South Africa; 50 Department of Physics, Carleton University, Ottawa, ON Canada; 51 Faculté des Sciences Ain Chock, Réseau Universitaire de Physique des Hautes Energies-Université Hassan II, Casablanca, Morocco; 52 Centre National de l’Energie des Sciences Techniques Nucleaires (CNESTEN), Rabat, Morocco; 53 Faculté des Sciences Semlalia, Université Cadi Ayyad, LPHEA-Marrakech, Marrakech, Morocco; 54 Faculté des Sciences, Université Mohamed Premier and LPTPM, Oujda, Morocco; 55 Faculté des sciences, Université Mohammed V, Rabat, Morocco; 56 CERN, Geneva, Switzerland; 57 Enrico Fermi Institute, University of Chicago, Chicago, IL USA; 58 LPC, Université Clermont Auvergne, CNRS/IN2P3, Clermont-Ferrand, France; 59 Nevis Laboratory, Columbia University, Irvington, NY USA; 60 Niels Bohr Institute, University of Copenhagen, Copenhagen, Denmark; 61 Dipartimento di Fisica, Università della Calabria, Rende, Italy; 62 INFN Gruppo Collegato di Cosenza, Laboratori Nazionali di Frascati, Frascati, Italy; 63 Physics Department, Southern Methodist University, Dallas, TX USA; 64 Physics Department, University of Texas at Dallas, Richardson, TX USA; 65 Department of Physics, Stockholm University, Stockholm, Sweden; 66 Oskar Klein Centre, Stockholm, Sweden; 67 Deutsches Elektronen-Synchrotron DESY, Hamburg and Zeuthen, Germany; 68 Lehrstuhl für Experimentelle Physik IV, Technische Universität Dortmund, Dortmund, Germany; 69 Institut für Kern- und Teilchenphysik, Technische Universität Dresden, Dresden, Germany; 70 Department of Physics, Duke University, Durham, NC USA; 71 SUPA-School of Physics and Astronomy, University of Edinburgh, Edinburgh, UK; 72 INFN e Laboratori Nazionali di Frascati, Frascati, Italy; 73 Physikalisches Institut, Albert-Ludwigs-Universität Freiburg, Freiburg, Germany; 74 II Physikalisches Institut, Georg-August-Universität Göttingen, Göttingen, Germany; 75 Département de Physique Nucléaire et Corpusculaire, Université de Genève, Geneva, Switzerland; 76 Dipartimento di Fisica, Università di Genova, Genoa, Italy; 77 INFN Sezione di Genova, Genoa, Italy; 78 II. Physikalisches Institut, Justus-Liebig-Universität Giessen, Giessen, Germany; 79 SUPA-School of Physics and Astronomy, University of Glasgow, Glasgow, UK; 80 LPSC, Université Grenoble Alpes, CNRS/IN2P3, Grenoble INP, Grenoble, France; 81 Laboratory for Particle Physics and Cosmology, Harvard University, Cambridge, MA USA; 82 Department of Modern Physics and State Key Laboratory of Particle Detection and Electronics, University of Science and Technology of China, Hefei, China; 83 Institute of Frontier and Interdisciplinary Science and Key Laboratory of Particle Physics and Particle Irradiation (MOE), Shandong University, Qingdao, China; 84 School of Physics and Astronomy, Shanghai Jiao Tong University, KLPPAC-MoE, SKLPPC, Shanghai, China; 85 Tsung-Dao Lee Institute, Shanghai, China; 86 Kirchhoff-Institut für Physik, Ruprecht-Karls-Universität Heidelberg, Heidelberg, Germany; 87 Physikalisches Institut, Ruprecht-Karls-Universität Heidelberg, Heidelberg, Germany; 88 Faculty of Applied Information Science, Hiroshima Institute of Technology, Hiroshima, Japan; 89 Department of Physics, Chinese University of Hong Kong, Shatin, N.T. Hong Kong; 90 Department of Physics, University of Hong Kong, Hong Kong, China; 91 Department of Physics and Institute for Advanced Study, Hong Kong University of Science and Technology, Clear Water Bay, Kowloon, Hong Kong, China; 92 Department of Physics, National Tsing Hua University, Hsinchu, Taiwan; 93 Department of Physics, Indiana University, Bloomington, IN USA; 94 INFN Gruppo Collegato di Udine, Sezione di Trieste, Udine, Italy; 95 ICTP, Trieste, Italy; 96 Dipartimento di Chimica, Fisica e Ambiente, Università di Udine, Udine, Italy; 97 INFN Sezione di Lecce, Lecce, Italy; 98 Dipartimento di Matematica e Fisica, Università del Salento, Lecce, Italy; 99 INFN Sezione di Milano, Milan, Italy; 100 Dipartimento di Fisica, Università di Milano, Milan, Italy; 101 INFN Sezione di Napoli, Naples, Italy; 102 Dipartimento di Fisica, Università di Napoli, Naples, Italy; 103 INFN Sezione di Pavia, Pavia, Italy; 104 Dipartimento di Fisica, Università di Pavia, Pavia, Italy; 105 INFN Sezione di Pisa, Pisa, Italy; 106 Dipartimento di Fisica E. Fermi, Università di Pisa, Pisa, Italy; 107 INFN Sezione di Roma, Rome, Italy; 108 Dipartimento di Fisica, Sapienza Università di Roma, Rome, Italy; 109 INFN Sezione di Roma Tor Vergata, Rome, Italy; 110 Dipartimento di Fisica, Università di Roma Tor Vergata, Rome, Italy; 111 INFN Sezione di Roma Tre, Rome, Italy; 112 Dipartimento di Matematica e Fisica, Università Roma Tre, Rome, Italy; 113 INFN-TIFPA, Trento, Italy; 114 Università degli Studi di Trento, Trento, Italy; 115 Institut für Astro- und Teilchenphysik, Leopold-Franzens-Universität, Innsbruck, Austria; 116 University of Iowa, Iowa City, IA USA; 117 Department of Physics and Astronomy, Iowa State University, Ames, IA USA; 118 Joint Institute for Nuclear Research, Dubna, Russia; 119 Departamento de Engenharia Elétrica, Universidade Federal de Juiz de Fora (UFJF), Juiz de Fora, Brazil; 120 Universidade Federal do Rio De Janeiro COPPE/EE/IF, Rio de Janeiro, Brazil; 121 Universidade Federal de São João del Rei (UFSJ), São João del Rei, Brazil; 122 Instituto de Física, Universidade de São Paulo, São Paulo, Brazil; 123 KEK, High Energy Accelerator Research Organization, Tsukuba, Japan; 124 Graduate School of Science, Kobe University, Kobe, Japan; 125 Faculty of Physics and Applied Computer Science, AGH University of Science and Technology, Kraków, Poland; 126 Marian Smoluchowski Institute of Physics, Jagiellonian University, Kraków, Poland; 127 Institute of Nuclear Physics Polish Academy of Sciences, Kraków, Poland; 128 Faculty of Science, Kyoto University, Kyoto, Japan; 129 Kyoto University of Education, Kyoto, Japan; 130 Research Center for Advanced Particle Physics and Department of Physics, Kyushu University, Fukuoka, Japan; 131 Instituto de Física La Plata, Universidad Nacional de La Plata and CONICET, La Plata, Argentina; 132 Physics Department, Lancaster University, Lancaster, UK; 133 Oliver Lodge Laboratory, University of Liverpool, Liverpool, UK; 134 Department of Experimental Particle Physics, Jožef Stefan Institute and Department of Physics, University of Ljubljana, Ljubljana, Slovenia; 135 School of Physics and Astronomy, Queen Mary University of London, London, UK; 136 Department of Physics, Royal Holloway University of London, Egham, UK; 137 Department of Physics and Astronomy, University College London, London, UK; 138 Louisiana Tech University, Ruston, LA USA; 139 Fysiska institutionen, Lunds Universitet, Lund, Sweden; 140 Centre de Calcul de l’Institut National de Physique Nucléaire et de Physique des Particules (IN2P3), Villeurbanne, France; 141 Departamento de Física Teorica C-15 and CIAFF, Universidad Autónoma de Madrid, Madrid, Spain; 142 Institut für Physik, Universität Mainz, Mainz, Germany; 143 School of Physics and Astronomy, University of Manchester, Manchester, UK; 144 CPPM, Aix-Marseille Université, CNRS/IN2P3, Marseille, France; 145 Department of Physics, University of Massachusetts, Amherst, MA USA; 146 Department of Physics, McGill University, Montreal, QC Canada; 147 School of Physics, University of Melbourne, Victoria, Australia; 148 Department of Physics, University of Michigan, Ann Arbor, MI USA; 149 Department of Physics and Astronomy, Michigan State University, East Lansing, MI USA; 150 B.I. Stepanov Institute of Physics, National Academy of Sciences of Belarus, Minsk, Belarus; 151 Research Institute for Nuclear Problems of Byelorussian State University, Minsk, Belarus; 152 Group of Particle Physics, University of Montreal, Montreal, QC Canada; 153 P.N. Lebedev Physical Institute of the Russian Academy of Sciences, Moscow, Russia; 154 Institute for Theoretical and Experimental Physics (ITEP), Moscow, Russia; 155 National Research Nuclear University MEPhI, Moscow, Russia; 156 D.V. Skobeltsyn Institute of Nuclear Physics, M.V. Lomonosov Moscow State University, Moscow, Russia; 157 Fakultät für Physik, Ludwig-Maximilians-Universität München, Munich, Germany; 158 Max-Planck-Institut für Physik (Werner-Heisenberg-Institut), Munich, Germany; 159 Nagasaki Institute of Applied Science, Nagasaki, Japan; 160 Graduate School of Science and Kobayashi-Maskawa Institute, Nagoya University, Nagoya, Japan; 161 Department of Physics and Astronomy, University of New Mexico, Albuquerque, NM USA; 162 Institute for Mathematics, Astrophysics and Particle Physics, Radboud University Nijmegen/Nikhef, Nijmegen, The Netherlands; 163 Nikhef National Institute for Subatomic Physics, University of Amsterdam, Amsterdam, The Netherlands; 164 Department of Physics, Northern Illinois University, DeKalb, IL USA; 165 Budker Institute of Nuclear Physics and NSU, SB RAS, Novosibirsk, Russia; 166 Novosibirsk State University, Novosibirsk, Russia; 167 Institute for High Energy Physics of the National Research Centre Kurchatov Institute, Protvino, Russia; 168 Department of Physics, New York University, New York, NY USA; 169 Ohio State University, Columbus, OH USA; 170 Faculty of Science, Okayama University, Okayama, Japan; 171 Homer L. Dodge Department of Physics and Astronomy, University of Oklahoma, Norman, OK USA; 172 Department of Physics, Oklahoma State University, Stillwater, OK USA; 173 Palacký University, RCPTM, Joint Laboratory of Optics, Olomouc, Czech Republic; 174 Center for High Energy Physics, University of Oregon, Eugene, OR USA; 175 LAL, Université Paris-Sud, CNRS/IN2P3, Université Paris-Saclay, Orsay, France; 176 Graduate School of Science, Osaka University, Osaka, Japan; 177 Department of Physics, University of Oslo, Oslo, Norway; 178 Department of Physics, Oxford University, Oxford, UK; 179 LPNHE, Sorbonne Université, Paris Diderot Sorbonne Paris Cité, CNRS/IN2P3 Paris, France; 180 Department of Physics, University of Pennsylvania, Philadelphia, PA USA; 181 Konstantinov Nuclear Physics Institute of National Research Centre “Kurchatov Institute”, PNPI, St. Petersburg, Russia; 182 Department of Physics and Astronomy, University of Pittsburgh, Pittsburgh, PA USA; 183 Laboratório de Instrumentação e Física Experimental de Partículas-LIP, Lisbon, Portugal; 184 Departamento de Física, Faculdade de Ciências, Universidade de Lisboa, Lisbon, Portugal; 185 Departamento de Física, Universidade de Coimbra, Coimbra, Portugal; 186 Centro de Física Nuclear da Universidade de Lisboa, Lisbon, Portugal; 187 Departamento de Física, Universidade do Minho, Braga, Portugal; 188 Departamento de Física Teorica y del Cosmos, Universidad de Granada, Granada, Spain; 189 Dep Física and CEFITEC of Faculdade de Ciências e Tecnologia, Universidade Nova de Lisboa, Caparica, Portugal; 190 Institute of Physics, Academy of Sciences of the Czech Republic, Prague, Czech Republic; 191 Czech Technical University in Prague, Prague, Czech Republic; 192 Faculty of Mathematics and Physics, Charles University, Prague, Czech Republic; 193 Particle Physics Department, Rutherford Appleton Laboratory, Didcot, UK; 194 IRFU, CEA , Université Paris-Saclay, Gif-sur-Yvette, France; 195 Santa Cruz Institute for Particle Physics, University of California Santa Cruz, Santa Cruz, CA USA; 196 Departamento de Física, Pontificia Universidad Católica de Chile, Santiago, Chile; 197 Departamento de Física, Universidad Técnica Federico Santa María, Valparaiso, Chile; 198 Department of Physics, University of Washington, Seattle, WA USA; 199 Department of Physics and Astronomy, University of Sheffield, Sheffield, UK; 200 Department of Physics, Shinshu University, Nagano, Japan; 201 Department Physik, Universität Siegen, Siegen, Germany; 202 Department of Physics, Simon Fraser University, Burnaby, BC Canada; 203 SLAC National Accelerator Laboratory, Stanford, CA USA; 204 Physics Department, Royal Institute of Technology, Stockholm, Sweden; 205 Departments of Physics and Astronomy, Stony Brook University, Stony Brook, NY USA; 206 Department of Physics and Astronomy, University of Sussex, Brighton, UK; 207 School of Physics, University of Sydney, Sydney, Australia; 208 Institute of Physics, Academia Sinica, Taipei, Taiwan; 209 Academia Sinica Grid Computing, Institute of Physics, Academia Sinica, Taipei, Taiwan; 210 E. Andronikashvili Institute of Physics, Iv. Javakhishvili Tbilisi State University, Tbilisi, Georgia; 211 High Energy Physics Institute, Tbilisi State University, Tbilisi, Georgia; 212 Department of Physics, Technion, Israel Institute of Technology, Haifa, Israel; 213 Raymond and Beverly Sackler School of Physics and Astronomy, Tel Aviv University, Tel Aviv, Israel; 214 Department of Physics, Aristotle University of Thessaloniki, Thessaloniki, Greece; 215 International Center for Elementary Particle Physics and Department of Physics, University of Tokyo, Tokyo, Japan; 216 Graduate School of Science and Technology, Tokyo Metropolitan University, Tokyo, Japan; 217 Department of Physics, Tokyo Institute of Technology, Tokyo, Japan; 218 Tomsk State University, Tomsk, Russia; 219 Department of Physics, University of Toronto, Toronto, ON Canada; 220 TRIUMF, Vancouver, BC Canada; 221 Department of Physics and Astronomy, York University, Toronto, ON Canada; 222 Division of Physics and Tomonaga Center for the History of the Universe, Faculty of Pure and Applied Sciences, University of Tsukuba, Tsukuba, Japan; 223 Department of Physics and Astronomy, Tufts University, Medford, MA USA; 224 Department of Physics and Astronomy, University of California Irvine, Irvine, CA USA; 225 Department of Physics and Astronomy, University of Uppsala, Uppsala, Sweden; 226 Department of Physics, University of Illinois, Urbana, IL USA; 227 Instituto de Física Corpuscular (IFIC), Centro Mixto Universidad de Valencia - CSIC, Valencia, Spain; 228 Department of Physics, University of British Columbia, Vancouver, BC Canada; 229 Department of Physics and Astronomy, University of Victoria, Victoria, BC Canada; 230 Fakultät für Physik und Astronomie, Julius-Maximilians-Universität Würzburg, Würzburg, Germany; 231 Department of Physics, University of Warwick, Coventry, UK; 232 Waseda University, Tokyo, Japan; 233 Department of Particle Physics, Weizmann Institute of Science, Rehovot, Israel; 234 Department of Physics, University of Wisconsin, Madison, WI USA; 235 Fakultät für Mathematik und Naturwissenschaften, Fachgruppe Physik, Bergische Universität Wuppertal, Wuppertal, Germany; 236 Department of Physics, Yale University, New Haven, CT USA; 237 Yerevan Physics Institute, Yerevan, Armenia; 238 CERN, 1211 Geneva 23, Switzerland

## Abstract

A search for the electroweak production of charginos, neutralinos and sleptons decaying into final states involving two or three electrons or muons is presented. The analysis is based on 36.1 fb$^{-1}$ of $\sqrt{s}=13$ TeV proton–proton collisions recorded by the ATLAS detector at the Large Hadron Collider. Several scenarios based on simplified models are considered. These include the associated production of the next-to-lightest neutralino and the lightest chargino, followed by their decays into final states with leptons and the lightest neutralino via either sleptons or Standard Model gauge bosons; direct production of chargino pairs, which in turn decay into leptons and the lightest neutralino via intermediate sleptons; and slepton pair production, where each slepton decays directly into the lightest neutralino and a lepton. No significant deviations from the Standard Model expectation are observed and stringent limits at 95% confidence level are placed on the masses of relevant supersymmetric particles in each of these scenarios. For a massless lightest neutralino, masses up to 580 GeV are excluded for the associated production of the next-to-lightest neutralino and the lightest chargino, assuming gauge-boson mediated decays, whereas for slepton-pair production masses up to 500 GeV are excluded assuming three generations of mass-degenerate sleptons.

## Introduction

Supersymmetry (SUSY) [1–7] is one of the most studied extensions of the Standard Model (SM). In its minimal realization (the Minimal Supersymmetric Standard Model, or MSSM) [8, 9], it predicts new fermionic (bosonic) partners of the fundamental SM bosons (fermions) and an additional Higgs doublet. These new SUSY particles, or sparticles, can provide an elegant solution to the gauge hierarchy problem [10–13]. In *R*-parity-conserving models [14], sparticles can only be produced in pairs and the lightest supersymmetric particle (LSP) is stable. This is typically assumed to be the $\displaystyle \tilde{\chi }^0_1$ neutralino,^1^ which can then provide a natural candidate for dark matter [15, 16]. If produced in proton–proton collisions, a neutralino LSP would escape detection and lead to an excess of events with large missing transverse momentum above the expectations from SM processes, a characteristic that is exploited to search for SUSY signals in analyses presented in this paper.

The production cross-sections of SUSY particles at the Large Hadron Collider (LHC) [17] depend both on the type of interaction involved and on the masses of the sparticles. The coloured sparticles (squarks and gluinos) are produced in strong interactions with significantly larger production cross-sections than non-coloured sparticles of equal masses, such as the charginos ($\tilde{\chi }^{\pm }_{i}$, $i = 1, 2$) and neutralinos ($\tilde{\chi }^{0}_{j}$, $j = 1, 2, 3, 4$) and the sleptons ($\tilde{\ell }$ and $\tilde{\nu }$). The direct production of charginos and neutralinos or slepton pairs can dominate SUSY production at the LHC if the masses of the gluinos and the squarks are significantly larger. With searches performed by the ATLAS [18] and CMS [19] experiments during LHC Run 2, the exclusion limits on coloured sparticle masses extend up to approximately $2\,$TeV [20–22], making electroweak production an increasingly important probe for SUSY signals at the LHC.

This paper presents a set of searches for the electroweak production of charginos, neutralinos and sleptons decaying into final states with two or three electrons or muons using 36.1 fb$^{-1}$ of proton–proton collision data delivered by the LHC at a centre-of-mass energy of $\sqrt{s}=13$ TeV. The results build on studies performed during LHC Run 1 at $\sqrt{s}=7$ TeV and 8 TeV by the ATLAS Collaboration [23–25]. Analogous studies by the CMS Collaboration are presented in Refs. [26–29].

After descriptions of the SUSY scenarios considered (Sect. 2), the experimental apparatus (Sect. 3), the simulated samples (Sect. 4) and the event reconstruction (Sect. 5), the analysis search strategy is discussed in Sect. 6. This is followed by Sect. 7, which describes the estimation of SM contributions to the measured yields in the signal regions, and by Sect. 8, which discusses systematic uncertainties affecting the searches. Results are presented in Sect. 9, together with the statistical tests used to interpret them in the context of relevant SUSY benchmark scenarios. Section 10 summarizes the main conclusions.

## SUSY scenarios and search strategy

**Fig. 1:**
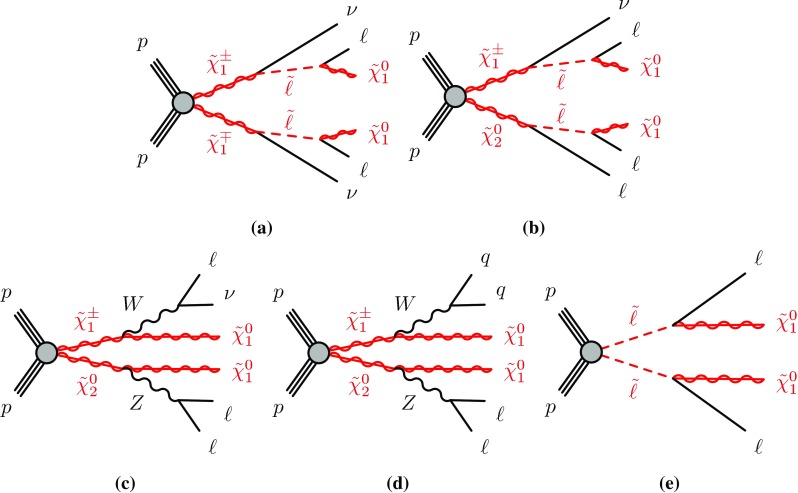
Diagrams of physics scenarios studied in this paper: **a** $\displaystyle \tilde{\chi }^+_1 \displaystyle \tilde{\chi }^-_1$ production with $\tilde{\ell }$-mediated decays into final states with two leptons, **b** $\displaystyle \tilde{\chi }^\pm _1 \displaystyle \tilde{\chi }^0_2$ production with $\tilde{\ell }$-mediated decays into final states with three leptons, **c** $\displaystyle \tilde{\chi }^\pm _1 \displaystyle \tilde{\chi }^0_2$ production with decays via leptonically decaying *W* and *Z* bosons into final states with three leptons, **d** $\displaystyle \tilde{\chi }^\pm _1 \displaystyle \tilde{\chi }^0_2$ production with decays via a hadronically decaying *W* boson and a leptonically decaying *Z* boson into final states with two leptons and two jets, and **e** slepton pair production with decays into final states with two leptons

This paper presents searches for the direct pair-production of $\displaystyle \tilde{\chi }^+_1 \displaystyle \tilde{\chi }^-_1$, $\displaystyle \tilde{\chi }^\pm _1 \displaystyle \tilde{\chi }^0_2$ and $\tilde{\ell } \tilde{\ell }$ particles, in final states with exactly two or three electrons and muons, two $\displaystyle \tilde{\chi }^0_1$ particles, and possibly additional jets or neutrinos. Simplified models [30], in which the masses of the relevant sparticles are the only free parameters, are used for interpretation and to guide the design of the searches. The pure wino $\displaystyle \tilde{\chi }^\pm _1$ and $\displaystyle \tilde{\chi }^0_2$ are taken to be mass-degenerate, and so are the scalar partners of the left-handed charged leptons and neutrinos ($\tilde{e}_{L }$, $\tilde{\mu }_{L }$, $\tilde{\tau }_{L }$ and $\tilde{\nu }$). Intermediate slepton masses, when relevant, are chosen to be midway between the mass of the heavier chargino and neutralino and that of the lightest neutralino, which is pure bino, and equal branching ratios for the three slepton flavours are assumed. The analysis sensitivity is not expected to depend strongly on the slepton mass hypothesis for a broad range of slepton masses, while it degrades as the slepton mass approaches that of the heavier chargino and neutralino, leading to lower $p_T$ values for the leptons produced in the heavy chargino and neutralino decays [25]. Lepton flavour is conserved in all models. Diagrams of processes considered are shown in Fig. 1. For models exploring $\displaystyle \tilde{\chi }^+_1 \displaystyle \tilde{\chi }^-_1$ production, it is assumed that the sleptons are also light and thus accessible in the sparticle decay chains, as illustrated in Fig. 1a. Two different classes of models are considered for $\displaystyle \tilde{\chi }^\pm _1 \displaystyle \tilde{\chi }^0_2$ production: in one case, the $\displaystyle \tilde{\chi }^\pm _1$ chargino and $\displaystyle \tilde{\chi }^0_2$ neutralino can decay into final-state SM particles and a $\displaystyle \tilde{\chi }^0_1$ neutralino via an intermediate $\tilde{\ell } _{L }$ or $\tilde{\nu }_{L }$, with a branching ratio of 50% to each (Fig. 1b); in the other case the $\displaystyle \tilde{\chi }^\pm _1$ chargino and $\displaystyle \tilde{\chi }^0_2$ neutralino decays proceed via SM gauge bosons (*W* or *Z*). For the gauge-boson-mediated decays, two distinct final states are considered: three-lepton (where lepton refers to an electron or muon) events where both the *W* and *Z* bosons decay leptonically (Fig. 1c) or events with two opposite-sign leptons and two jets where the *W* boson decays hadronically and the *Z* boson decays leptonically (Fig. 1d). In models with direct $\tilde{\ell } \tilde{\ell }$ production, each slepton decays into a lepton and a $\displaystyle \tilde{\chi }^0_1$ with a 100% branching ratio (Fig. 1e), and $\tilde{e}_{L }$, $\tilde{e}_{R }$, $\tilde{\mu }_{L }$, $\tilde{\mu }_{R }$, $\tilde{\tau }_{L }$ and $\tilde{\tau }_{R }$ are assumed to be mass-degenerate.

Events are recorded using triggers requiring the presence of at least two leptons and assigned to one of three mutually exclusive analysis channels depending on the lepton and jet multiplicity. The 2$\ell$ + 0 jets channel targets chargino- and slepton-pair production, the 2$\ell$ + jets channel targets chargino-neutralino production with gauge-boson-mediated decays, and the 3$\ell$ channel targets chargino-neutralino production with slepton- or gauge-boson-mediated decays. For each channel, a set of signal regions (SR), defined in Section 6, use requirements on $E_{\text {T}}^{\text {miss}}$ and other kinematic quantities, which are optimized for different SUSY models and sparticle masses. The analyses employ “inclusive” SRs to quantify significance without assuming a particular signal model and to exclude regions of SUSY model parameter space, as well as sets of orthogonal “exclusive” SRs that are considered simultaneously during limit-setting to improve the exclusion sensitivity.

## ATLAS detector

The ATLAS experiment [18] is a multi-purpose particle detector with a forward-backward symmetric cylindrical geometry and nearly $4\pi$ coverage in solid angle.^2^ The interaction point is surrounded by an inner detector (ID), a calorimeter system, and a muon spectrometer.

The ID provides precision tracking of charged particles for pseudorapidities $|\eta | < 2.5$ and is surrounded by a superconducting solenoid providing a 2 T axial magnetic field. The ID consists of silicon pixel and microstrip detectors inside a transition radiation tracker. One significant upgrade for the $\sqrt{s}=13$ TeV running period is the installation of the insertable B-layer [31], an additional pixel layer close to the interaction point which provides high-resolution hits at small radius to improve the tracking performance.

In the pseudorapidity region $|\eta | < 3.2$, high-granularity lead/liquid-argon (LAr) electromagnetic (EM) sampling calorimeters are used. A steel/scintillator tile calorimeter measures hadron energies for $|\eta | < 1.7$. The endcap and forward regions, spanning $1.5<|\eta | <4.9$, are instrumented with LAr calorimeters, for both the EM and hadronic measurements.

The muon spectrometer consists of three large superconducting toroids with eight coils each, and a system of trigger and precision-tracking chambers, which provide triggering and tracking capabilities in the ranges $|\eta | < 2.4$ and $|\eta | < 2.7$, respectively.

A two-level trigger system is used to select events [32]. The first-level trigger is implemented in hardware and uses a subset of the detector information. This is followed by the software-based high-level trigger, which runs offline reconstruction and calibration software, reducing the event rate to about 1 kHz.

## Data and simulated event samples

This analysis uses proton–proton collision data delivered by the LHC at $\sqrt{s}=13 \text { TeV}$ in 2015 and 2016. After fulfilling data-quality requirements, the data sample amounts to an integrated luminosity of 36.1 fb$^{-1}$. This value is derived using a methodology similar to that detailed in Refs. [33], from a calibration of the luminosity scale using *x*–*y* beam-separation scans performed in August 2015 and May 2016.

Various samples of Monte Carlo (MC) simulated events are used to model the SUSY signal and help in the estimation of the SM backgrounds. The samples include an ATLAS detector simulation [34], based on Geant4 [35], or a fast simulation [34] that uses a parameterization of the calorimeter response [36] and Geant4 for the other parts of the detector. The simulated events are reconstructed in the same manner as the data.

Diboson processes were simulated with the Sherpa  v2.2.1 event generator [37, 38] and normalized using next-to-leading-order (NLO) cross-sections [39, 40]. The matrix elements containing all diagrams with four electroweak vertices with additional hard parton emissions were calculated with Comix [41] and virtual QCD corrections were calculated with OpenLoops [42]. Matrix element calculations were merged with the Sherpa parton shower [43] using the ME+PS@NLO prescription [44]. The NNPDF3.0 NNLO parton distribution function (PDF) set [45] was used in conjunction with dedicated parton shower tuning developed by the Sherpa authors. The fully leptonic channels were calculated at NLO in the strong coupling constant with up to one additional parton for $4\ell$ and $2\ell +2\nu$, at NLO with no additional parton for $3\ell +\nu$, and at leading order (LO) with up to three additional partons. Processes with one of the bosons decaying hadronically and the other leptonically were calculated with up to one additional parton at NLO and up to three additional partons at LO.

Diboson processes with six electroweak vertices, such as same-sign *W* boson production in association with two jets, $W^\pm W^\pm jj$, and triboson processes were simulated as above with Sherpa  v2.2.1 using the NNPDF3.0 PDF set. Diboson processes with six vertices were calculated at LO with up to one additional parton. Fully leptonic triboson processes (*WWW*, *WWZ*, *WZZ* and *ZZZ*) were calculated at LO with up to two additional partons and at NLO for the inclusive processes and normalized using NLO cross-sections.

Events containing $$Z$$ bosons and associated jets ($$Z/\gamma ^*$$ + jets, also referred to as *Z* + jets in the following) were also produced using the Sherpa v2.2.1 generator with massive *b* / *c*-quarks to improve the treatment of the associated production of $$Z $$ bosons with jets containing *b*- and *c*-hadrons [46]. Matrix elements were calculated with up to two additional partons at NLO and up to four additional partons at LO, using Comix, OpenLoops, and Sherpa parton shower with ME+PS@NLO in a way similar to that described above. A global *K*-factor was used to normalize the *Z* + jets events to the next-to-next-to-leading-order (NNLO) QCD cross-sections [47].

For the production of $t\bar{t}$ and single top quarks in the *Wt* channel, the Powheg-Box v2 [48, 49] generator with the CT10 PDF set [50] was used, as discussed in Ref. [51]. The top quark mass was set at 172.5 GeV for all MC samples involving top quark production. The $t\bar{t}$ events were normalized using the NNLO+next-to-next-to-leading-logarithm (NNLL) QCD [52] cross-section, while the cross-section for single-top-quark events was calculated at NLO+NNLL [53].

Samples of $t\bar{t} V$ (with $V=W$ and *Z*, including non-resonant $Z/\gamma ^*$ contributions) and $t\bar{t}WW$ production were generated at LO with MadGraph5_aMC@NLO v2.2.2 [54] interfaced to Pythia 8.186 [55] for parton showering, hadronisation and the description of the underlying event, with up to two ($t\bar{t} W$), one ($t\bar{t} Z$) or no ($t\bar{t} WW$) extra partons included in the matrix element, as described in Ref. [56]. MadGraph was also used to simulate the *tZ*, $t\bar{t} t\bar{t}$ and $t\bar{t} t$ processes. A set of tuned parameters called the A14 tune [57] was used together with the NNPDF2.3LO PDF set [58]. The $t\bar{t} W$, $t\bar{t} Z$, $t\bar{t} WW$ and $t\bar{t} t\bar{t}$ events were normalized using their NLO cross-section [56] while the generator cross-section was used for *tZ* and $t\bar{t} t$.

Higgs boson production processes (including gluon–gluon fusion, associated *VH* production and vector-boson fusion) were generated using Powheg-Box v2 [59] and Pythia 8.186 and normalized using cross-sections calculated at NNLO with soft gluon emission effects added at NNLL accuracy [60], whilst $t\bar{t} H$ events were produced using MadGraph5_aMC@NLO 2.3.2 + Herwig++  [61] and normalized using the NLO cross-section [56]. All samples assume a Higgs boson mass of 125 GeV.

The SUSY signal processes were generated from LO matrix elements with up to two extra partons, using the MadGraph v2.2.3 generator interfaced to Pythia 8.186 with the A14 tune for the modelling of the SUSY decay chain, parton showering, hadronization and the description of the underlying event. Parton luminosities were provided by the NNPDF2.3LO PDF set. Jet–parton matching was realized following the CKKW-L prescription [62], with a matching scale set to one quarter of the pair-produced superpartner mass. Signal cross-sections were calculated at NLO, with soft gluon emission effects added at next-to-leading-logarithm (NLL) accuracy [63–67]. The nominal cross-section and its uncertainty were taken from an envelope of cross-section predictions using different PDF sets and factorization and renormalization scales, as described in Ref. [68]. The cross-section for $\displaystyle \tilde{\chi }^+_1 \displaystyle \tilde{\chi }^-_1$ production, each with a mass of 600 GeV, is $9.50\pm 0.91$ fb, while the cross-section for $\displaystyle \tilde{\chi }^\pm _1 \displaystyle \tilde{\chi }^0_2$ production, each with a mass of 800 GeV, is $4.76\pm 0.56$ fb.

In all MC samples, except those produced by Sherpa, the EvtGen v1.2.0 program [69] was used to model the properties of *b*- and *c*-hadron decays. To simulate the effects of additional *pp* collisions per bunch crossing (pile-up), additional interactions were generated using the soft QCD processes of Pythia 8.186 with the A2 tune [70] and the MSTW2008LO PDF set [71], and overlaid onto the simulated hard-scatter event. The Monte Carlo samples were reweighted so that the distribution of the number of pile-up interactions matches the distribution in data.

## Event reconstruction and preselection

Events used in the analysis were recorded during stable data-taking conditions and must have a reconstructed primary vertex [72] with at least two associated tracks with $p_{\text {T}} >400~\hbox {MeV}$. The primary vertex of an event is identified as the vertex with the highest $\Sigma p_{\text {T}} ^2$ of associated tracks.

Two identification criteria are defined for the objects used in these analyses, referred to as “baseline” and “signal” (with the signal objects being a subset of the baseline ones). The former are defined to disambiguate between overlapping physics objects and to perform data-driven estimations of non-prompt leptonic backgrounds (discussed in Sect. 7) while the latter are used to construct kinematic and multiplicity discriminating variables needed for the event selection.

Baseline electrons are reconstructed from isolated electromagnetic calorimeter energy deposits matched to ID tracks and are required to have $|\eta |<2.47$, $p_{\text {T}} >{10}\,{\text {GeV}}$, and to pass a loose likelihood-based identification requirement [73, 74]. The likelihood input variables include measurements of calorimeter shower shapes and track properties from the ID.

Baseline muons are reconstructed in the region $|\eta |<2.7$ from muon spectrometer tracks matching ID tracks. All muons must have $p_{\text {T}} >{10}\,{\text {GeV}}$ and must pass the “medium identification” requirements defined in Ref. [75], based on selection of the number of hits and curvature measurements in the ID and muon spectrometer systems.

Jets are reconstructed with the anti-$k_t$ algorithm [76] as implemented in the FastJet package [77], with radius parameter $R=0.4$, using three-dimensional energy clusters in the calorimeter [78] as input. Baseline jets must have $p_{\text {T}} >{20}\,{\text {GeV}}$ and $|\eta |<4.5$ and signal jets have the tighter requirement of $|\eta |<2.4$. Jet energies are calibrated as described in Refs. [79, 80]. In order to reduce the effects of pile-up, jets with $p_{\text {T}} <{60}\,{\hbox {GeV}}$ and $|\eta |<2.4$ must have a significant fraction of their associated tracks compatible with originating from the primary vertex, as defined by the jet vertex tagger [81]. Furthermore, for all jets the expected average energy contribution from pile-up is subtracted according to the jet area [81, 82]. Events are discarded if they contain any jet that is judged by basic quality criteria to be detector noise or non-collision background.

Identification of jets containing *b*-hadrons (*b*-jets), so called *b*-tagging, is performed with the MV2c10 algorithm, a multivariate discriminant making use of track impact parameters and reconstructed secondary vertices [83, 84]. A requirement is chosen corresponding to a 77% average efficiency obtained for *b*-jets in simulated $t\bar{t}$ events. The corresponding rejection factors against jets originating from *c*-quarks, from $\tau$-leptons, and from light quarks and gluons in the same sample at this working point are 6, 22 and 134, respectively.

Baseline photon candidates are required to meet the “tight” selection criteria of Ref. [85] and satisfy $p_{\text {T}} >25$ GeV and $|\eta |<2.37$, but excluding the transition region $1.37<|\eta |<1.52$, where the calorimeter performance is degraded.

After object identification, an “object-removal procedure” is performed on all baseline objects to remove possible double-counting in the reconstruction:

1. Any electron sharing an ID track with a muon is removed.
2. If a *b*-tagged jet (identified using the 85% efficiency working point of the MV2c10 algorithm) is within $\Delta R = 0.2$ of an electron candidate, the electron is rejected, as it is likely to be from a semileptonic *b*-hadron decay; if the jet within $\Delta R = 0.2$ of the electron is not *b*-tagged, the jet itself is discarded, as it likely originates from an electron-induced shower.
3. Electrons within $\Delta R=0.4$ of a remaining jet candidate are discarded, to further suppress electrons from semileptonic decays of *b*- and *c*-hadrons.
4. Jets with a nearby muon that carries a significant fraction of the transverse momentum of the jet ($p_{\text {T}} ^\mu > 0.7 \sum p_{\text {T}} ^{\text {jet tracks}}$, where $p_{\text {T}} ^\mu$ and $p_{\text {T}} ^{\text {jet tracks}}$ are the transverse momenta of the muon and the tracks associated with the jet, respectively) are discarded either if the candidate muon is within $\Delta R=0.2$ of the jet or if the muon is matched to a track associated with the jet. Only jets with fewer than three associated tracks can be discarded in this step.
5. Muons within $\Delta R=0.4$ of a remaining jet candidate are discarded to suppress muons from semileptonic decays of *b*- and *c*-hadrons.

Signal electrons must satisfy a “medium” likelihood-based identification requirement [73] and the track associated with the electron must have a significance of the transverse impact parameter relative to the reconstructed primary vertex, $d_0$, of $\vert d_0\vert /\sigma (d_0) < 5$, with $\sigma (d_0)$ being the uncertainty in $d_0$. In addition, the longitudinal impact parameter (again relative to the reconstructed primary vertex), $z_0$, must satisfy $\vert z_0 \sin \theta \vert < 0.5$ mm. Similarly, signal muons must satisfy the requirements of $\vert d_0\vert /\sigma (d_0) < 3$, $\vert z_0 \sin \theta \vert < 0.5$ mm, and additionally have $|\eta |<2.4$. Isolation requirements are also applied to both the signal electrons and muons to reduce the contributions of “fake” or non-prompt leptons, which originate from misidentified hadrons, photons conversions, and hadron decays. These $p_{\text {T}}$- and $\eta$-dependent requirements use track- and calorimeter-based information and have efficiencies in $Z\rightarrow e^{+} e^{-}$ and $Z\rightarrow \mu ^{+} \mu ^{-}$ events that rise from 95% at 25 GeV to 99% at 60 GeV.

The missing transverse momentum $\mathbf {p}_\mathrm {T}^\mathrm {miss}$, with magnitude $E_{\text {T}}^{\text {miss}}$, is the negative vector sum of the transverse momenta of all identified physics objects (electrons, photons, muons, jets) and an additional soft term. The soft term is constructed from all tracks that are not associated with any physics object and that are associated with the primary vertex, to suppress contributions from pile-up interactions. The $E_{\text {T}}^{\text {miss}}$ value is adjusted for the calibration of the jets and the other identified physics objects above [86].

Events considered in the analysis must pass a trigger selection requiring either two electrons, two muons or an electron plus a muon. The trigger-level thresholds on the $p_{\text {T}}$ value of the leptons involved in the trigger decision are in the range 8–22 GeV and are looser than those applied offline to ensure that trigger efficiencies are constant in the relevant phase space.

Events containing a photon and jets are used to estimate the *Z* + jets background in events with two leptons and jets. These events are selected with a set of prescaled single-photon triggers with $p_{\text {T}}$ thresholds in the range 35–100 GeV and an unprescaled single-photon trigger with threshold $p_{\text {T}} =140$ GeV. Signal photons in this control sample must have $p_{\text {T}} >37$ GeV to be in the efficiency plateau of the lowest-threshold single-photon trigger, fall outside the barrel-endcap transition region defined by $1.37< |\eta | < 1.52$, and pass “tight” selection criteria described in Ref. [87], as well as $p_{\text {T}}$- and $\eta$-dependent requirements on both track- and calorimeter-based isolation.

Simulated events are corrected to account for small differences in the signal lepton trigger, reconstruction, identification, isolation, as well as *b*-tagging efficiencies between data and MC simulation.

## Signal regions

In order to search for the electroweak production of supersymmetric particles, three different search channels that target different SUSY processes are defined:

- 2$\ell$+ 0 jets channel: targets $\displaystyle \tilde{\chi }^+_1 \displaystyle \tilde{\chi }^-_1$ and $\tilde{\ell } \tilde{\ell }$ production (shown in Fig. 1a, e) in signal regions with a jet veto and defined using the “stransverse mass” variable, $m_{\mathrm{T2}}$ [88, 89], and the dilepton invariant mass $m_{\ell \ell }$;
- 2$\ell$+ jets channel: targets $\displaystyle \tilde{\chi }^\pm _1 \displaystyle \tilde{\chi }^0_2$ production with decays via gauge bosons (shown in Fig. 1d) into two same-flavour opposite-sign (SFOS) leptons (from the *Z* boson) and at least two jets (from the *W* boson);
- 3$\ell$ channel: targets $\displaystyle \tilde{\chi }^\pm _1 \displaystyle \tilde{\chi }^0_2$ production with decays via intermediate $\tilde{\ell }$ or gauge bosons into three-lepton final states (shown in Fig. 1b, c).

In each channel, inclusive and/or exclusive signal regions (SRs) are defined that require exactly two or three signal leptons, with vetos on any additional baseline leptons. In the 2$\ell$ + 0 jets channel only, this additional baseline lepton veto is applied before considering overlap-removal. The leading and sub-leading leptons are required to have $p_{\text {T}}$ $>25$ GeV and 20 GeV respectively; however, in the 2$\ell$ + jets and 3$\ell$ channels, tighter lepton $p_{\text {T}}$ requirements are applied to the sub-leading leptons.

### Signal regions for 2$\ell$ + 0 jets channel

In the 2$\ell$ + 0 jets channel the leptons are required to be of opposite sign and events are separated into “same flavour” (SF) events (corresponding to dielectron, $e^{+}e^{-}$, and dimuon, $\mu ^{+}\mu ^{-}$, events) and “different flavour” (DF) events (electron–muon, $e^{\pm }\mu ^{\mp }$). This division is driven by the different background compositions in the two classes of events. All events used in the SRs are required to have a dilepton invariant mass $m_{\ell \ell }>40$ GeV and not contain any *b*-tagged jets with $p_{\text {T}}$ $>20$ GeV or non-*b*-tagged jets with $p_{\text {T}}$ $>60$ GeV.

After this preselection, exclusive signal regions are used to maximize exclusion sensitivity across the simplified model parameter space for $\displaystyle \tilde{\chi }^+_1 \displaystyle \tilde{\chi }^-_1$ and $\tilde{\ell } \tilde{\ell }$ production. In the SF regions a two-dimensional binning in $m_\mathrm {T2}$ and $m_{\ell \ell }$ is used as high-$m_{\ell \ell }$ requirements provide strong suppression of the *Z* + jets background, whereas in the DF regions, where the *Z* + jets background is negligible, a one-dimensional binning in $m_\mathrm {T2}$ is sufficient. The stransverse mass $m_\mathrm {T2}$ is defined as:

$$\begin{aligned} m_\mathrm {T2}= \min _{\mathbf {q}_\mathrm {T}}\left[ \max \left( m_\mathrm {T}(\mathbf {p}_\mathrm {T}^{\ell 1},\mathbf {q}_\mathrm {T}),m_\mathrm {T}(\mathbf {p}_\mathrm {T}^{\ell 2}, \mathbf {p}_\mathrm {T}^\mathrm {miss}-\mathbf {q}_\mathrm {T})\right) \right] , \end{aligned}$$

where $\mathbf {p}_\mathrm {T}^{\ell 1}$ and $\mathbf {p}_\mathrm {T}^{\ell 2}$ are the transverse momentum vectors of the two leptons, and $\mathbf {q}_\mathrm {T}$ is a transverse momentum vector that minimizes the larger of $m_\mathrm {T}(\mathbf {p}_\mathrm {T}^{\ell 1},\mathbf {q}_\mathrm {T})$ and $m_\mathrm {T}(\mathbf {p}_\mathrm {T}^{\ell 2},\mathbf {p}_\mathrm {T}^\mathrm {miss}-\mathbf {q}_\mathrm {T})$, where:

$$\begin{aligned} m_\mathrm {T}(\mathbf {p}_\mathrm {T},\mathbf {q}_\mathrm {T}) = \sqrt{2(p_{\text {T}} q_\mathrm {T}-\mathbf {p}_\mathrm {T}\cdot \mathbf {q}_\mathrm {T})}. \end{aligned}$$

For SM backgrounds of $t\bar{t}$ and *WW* production in which the missing transverse momentum and the pair of selected leptons originate from two $W\rightarrow \ell \nu$ decays and all momenta are accurately measured, the $m_\mathrm {T2}$ value must be less than the *W* boson mass $m_W$, and requiring the $m_\mathrm {T2}$ value to significantly exceed $m_W$ thus strongly suppresses these backgrounds while retaining high efficiency for many SUSY signals.

When producing model-dependent exclusion limits in the $\displaystyle \tilde{\chi }^+_1 \displaystyle \tilde{\chi }^-_1$ simplified models, all signal regions are statistically combined, whereas only the same-flavour regions are used when probing $\tilde{\ell } \tilde{\ell }$ production. In addition, a set of inclusive signal regions are also defined, and these are used to provide a more model-independent test for an excess of events. The definitions of both the exclusive and inclusive signal regions are provided in Table 1.

**Table 1:** The definitions of the exclusive and inclusive signal regions for the 2$\ell$ + 0 jets channel. Relevant kinematic variables are defined in the text. The bins labelled “DF”or “SF” refer to signal regions with different-flavour or same-flavour lepton pair combinations, respectively

$m_\mathrm {T2}$ [GeV]	$m_{\ell \ell }$ [GeV]	SF bin	DF bin
2$\ell$+ 0 jets exclusive signal region definitions			
100–150	111–150	SR2-SF-a	-
	150–200	SR2-SF-b	-
	200–300	SR2-SF-c	-
	$>300$	SR2-SF-d	-
	$>111$	-	SR2-DF-a
150–200	111–150	SR2-SF-e	-
	150–200	SR2-SF-f	-
	200–300	SR2-SF-g	-
	$>300$	SR2-SF-h	-
	$>111$	-	SR2-DF-b
200–300	111–150	SR2-SF-i	-
	150–200	SR2-SF-j	-
	200–300	SR2-SF-k	-
	$>300$	SR2-SF-l	-
	$>111$	-	SR2-DF-c
$>300$	$>111$	SR2-SF-m	SR2-DF-d
2$\ell +$ 0 jets inclusive signal region definitions			
> 100	> 111	SR2-SF-loose	–
> 130	> 300	SR2-SF-tight	–
> 100	> 111	–	SR2-DF-100
> 150	> 111	–	SR2-DF-150
> 200	> 111	–	SR2-DF-200
> 300	> 111	–	SR2-DF-300

### Signal regions for 2$\ell$ + jets channel

In the 2$\ell$ + jets channel, two inclusive signal regions differing only in the $E_{\text {T}}^{\text {miss}}$ requirement, denoted SR2-int and SR2-high, are used to target intermediate and large mass splittings between the $\displaystyle \tilde{\chi }^\pm _1/\displaystyle \tilde{\chi }^0_2$ chargino/neutralino and the $\displaystyle \tilde{\chi }^0_1$ neutralino. In addition to the preselection used in the 2$\ell$ + 0 jets channel, with the exception of the veto requirement on non-*b*-tagged jets, the sub-leading lepton is also required to have $p_{\text {T}}$ $>25$ GeV and events must have at least two jets, with the leading two jets satisfying $p_{\text {T}}$ $>30$ GeV. The *b*-jet veto is applied in the same way as in the 2$\ell$ + 0 jets channel. Several kinematic requirements are applied to select two leptons consistent with an on-shell *Z* boson and two jets consistent with a *W* boson. A tight requirement of $m_\mathrm {T2}>100$ GeV is used to suppress the $t\bar{t}$ and *WW* backgrounds and $E_{\text {T}}^{\text {miss}} >150~(250)$ GeV is required for SR2-int (SR2-high).

An additional region in the 2$\ell$ + jets channel, denoted SR2-low, is optimized for the region of parameter space where the mass splitting between the $\displaystyle \tilde{\chi }^\pm _1/\displaystyle \tilde{\chi }^0_2$ and the $\displaystyle \tilde{\chi }^0_1$ is similar to the *Z* boson mass and the signal becomes kinematically similar to the diboson (*VV*) backgrounds. It is split into two orthogonal subregions for performing background estimation and validation, and these are merged when presenting the results in Sect. 9. SR2-low-2J requires exactly two jets, with $p_{\text {T}} >30$ GeV, that are both assumed to originate from the *W* boson, while SR2-low-3J requires 3–5 signal jets (with the leading two jets satisfying $p_{\text {T}}$ $>30$ GeV) and assumes the $\displaystyle \tilde{\chi }^\pm _1 \displaystyle \tilde{\chi }^0_2$ system recoils against initial-state-radiation (ISR) jet(s). In the latter case, the two jets originating from the *W* boson are selected to be those closest in $\Delta \phi$ to the $Z(\rightarrow \ell \ell ) + E_{\text {T}}^{\text {miss}}$ system. This is different from SR2-int and SR2-high, where the two jets with the highest $p_{\text {T}}$ in the event are used to define the *W* boson candidate. The rest of the jets that are not associated with the *W* boson are collectively defined as ISR jets. All regions use variables, including angular distances and the *W* and *Z* boson transverse momenta, to select the signal topologies of interest. The definitions of the signal regions in the 2$\ell$ + jets channel are summarized in Table 2.

**Table 2:** Signal region definitions used for the 2$\ell$ + jets channel. Relevant kinematic variables are defined in the text. The symbols *W* and *Z* correspond to the reconstructed *W* and *Z* bosons in the final state. The *Z* boson is always reconstructed from the two leptons, whereas the *W* boson is reconstructed from the two jets leading in $p_{\text {T}}$ for SR2-int, SR2-high and the 2-jets channel of SR2-low, whilst for the 3–5 jets channel of SR2-low it is reconstructed from the two jets which are closest in $\Delta \phi$ to the *Z* ($\rightarrow \ell \ell$) + $E_{\text {T}}^{\text {miss}}$ system. The $\Delta R_{(jj)}$ and $m_{jj}$ variables are calculated using the two jets assigned to the *W* boson. ISR refers to the vectorial sum of the initial-state-radiation jets in the event (i.e. those not used in the reconstruction of the *W* boson) and jet1 and jet3 refer to the leading and third leading jet respectively. The variable $n_{\text {non-} b \text {-tagged jets}}$ refers to the number of jets with $p_{\text {T}} >30$ GeV that do not satisfy the *b*-tagging criteria

	SR2-int	SR2-high	SR2-low-2J	SR2-low-3J
2$\ell$ + jets signal region definitions				
$n_{\text {non-} b \text {-tagged jets}}$	$\ge 2$	$\ge 2$	2	3–5
$m_{\ell \ell }$ [GeV]	81–101	81–101	81–101	86–96
$m_{jj}$ [GeV]	70–100	70–100	70–90	70–90
$E_{\text {T}}^{\text {miss}}$ [GeV]	$>150$	$>250$	$>100$	$>100$
$p^{Z}_\mathrm{T}$ [GeV]	$>80$	$>80$	$>60$	$>40$
$p^{W}_\mathrm{T}$ [GeV]	$>100$	$>100$		
$m_\mathrm{T2}$ [GeV]	$>100$	$>100$		
$\Delta R_{(jj)}$	$<1.5$	$<1.5$		$<2.2$
$\Delta R_{(\ell \ell )}$	$<1.8$	$<1.8$		
$\Delta \phi _{(\mathbf {p}_\mathrm {T}^\mathrm {miss},Z)}$			$<0.8$	
$\Delta \phi _{(\mathbf {p}_\mathrm {T}^\mathrm {miss},W)}$	0.5–3.0	0.5–3.0	$>1.5$	$<2.2$
$E_{\text {T}}^{\text {miss}}/p^{Z}_\mathrm{T}$			0.6–1.6	
$E_{\text {T}}^{\text {miss}}/p^{W}_\mathrm{T}$			$<0.8$	
$\Delta \phi _{(\mathbf {p}_\mathrm {T}^\mathrm {miss},\mathrm{ISR})}$				$>2.4$
$\Delta \phi _{(\mathbf {p}_\mathrm {T}^\mathrm {miss},\mathrm{jet1})}$				$>2.6$
$E_{\text {T}}^{\text {miss}}/p^\mathrm{{ISR}}_\mathrm{T}$				0.4–0.8
$|\eta (Z)|$				$<1.6$
$p^{\mathrm{jet3}}_\mathrm{T}$ [GeV]				$>30$

### Signal regions for 3$\ell$ channel

The 3$\ell$ channel targets $\displaystyle \tilde{\chi }^\pm _1 \displaystyle \tilde{\chi }^0_2$ production and uses kinematic variables such as $E_{\text {T}}^{\text {miss}}$ and the transverse mass $m_\mathrm {T}$, which were used in the Run 1 analysis [24]. Events are required to have exactly three signal leptons and no additional baseline leptons, as well as zero *b*-tagged jets with $p_{\text {T}} >20$ GeV. In addition, two of the leptons must form an SFOS pair (as expected in $\displaystyle \tilde{\chi }^0_2 \rightarrow \ell ^{+}\ell ^{-}\displaystyle \tilde{\chi }^0_1$ decays). To resolve ambiguities when multiple SFOS pairings are present, the transverse mass is calculated using the unpaired lepton and $\mathbf {p}_\mathrm {T}^\mathrm {miss}$ for each possible SFOS pairing, and the lepton that yields the minimum transverse mass is assigned to the *W* boson. This transverse mass value is denoted by $m_{\mathrm{T}}^{\mathrm{min}}$, and is used alongside $E_{\text {T}}^{\text {miss}}$, jet multiplicity (in the gauge-boson-mediated scenario) and other relevant kinematic variables to define exclusive signal regions that have sensitivity to $\tilde{\ell }$-mediated and gauge-boson-mediated decays. The definitions of these exclusive regions are provided in Table 3. The bins denoted “slep-a,b,c,d,e” target $\tilde{\ell }$-mediated decays and consequently have a veto on SFOS pairs with an invariant mass consistent with the *Z* boson (this suppresses the *WZ* background). The invariant mass of the SFOS pair, $m_{\ell \ell }$, the magnitude of the missing transverse momentum, $E_{\text {T}}^{\text {miss}}$, and the $p_{\text {T}}$ value of the third leading lepton, $p_{\text {T}}^{\ell _{3}}$, are used to define the SR bins. Conversely, the bins denoted “WZ-0Ja,b,c” and ”WZ-1Ja,b,c” target gauge-boson-mediated decays and thus require the SFOS pair to have an invariant mass consistent with an on-shell *Z* boson. The 0-jet and $\ge$ 1-jet channels are considered separately and the regions are binned in $m_\mathrm {T}^{\mathrm{min}}$ and $E_{\text {T}}^{\text {miss}}$.

**Table 3:** Summary of the exclusive signal regions used in the 3$\ell$ channel. Relevant kinematic variables are defined in the text. The bins labelled “slep” target slepton-mediated decays whereas those labelled “WZ” target gauge-boson-mediated decays. The variable $n_{\text {non-} b \text {-tagged jets}}$ refers to the number of jets with $p_{\text {T}} >20$ GeV that do not satisfy the *b*-tagging criteria. Values of $p^{\ell _{3}}_\mathrm{T}$ refer to the $p_{\text {T}}$ of the third leading lepton and $p_{\text {T}} ^{\mathrm{jet1}}$ denotes the $p_{\text {T}}$ of the leading jet

$m_{\mathrm{SFOS}}$ [GeV]	$E_{\text {T}}^{\text {miss}}$ [GeV]	$p^{\ell _{3}}_\mathrm{T}$ [GeV]	$n_{\text {non-} b \text {-tagged jets}}$	$m_\mathrm {T}^{\mathrm{min}}$ [GeV]	$p^{\ell \ell \ell }_\mathrm{T}$ [GeV]	$p_{\text {T}} ^{jet1 }$ [GeV]	Bins
3$\ell$ exclusive signal region definitions							
$\le 81.2$	$>130$	20–30		$>110$			SR3-slep-a
	$>130$	$>30$		$>110$			SR3-slep-b
$\ge$ 101.2	$>130$	20–50		$>110$			SR3-slep-c
	$>130$	50–80		$>110$			SR3-slep-d
	$>130$	$>80$		$>110$			SR3-slep-e
81.2–101.2	60-120		0	$>110$			SR3-WZ-0Ja
	120–170		0	$>110$			SR3-WZ-0Jb
	$>170$		0	$>110$			SR3-WZ-0Jc
81.2–101.2	120–200		$\ge$ 1	$>110$	$<120$	$>70$	SR3-WZ-1Ja
	$>200$		$\ge$ 1	110–160			SR3-WZ-1Jb
	$>200$	$>35$	$\ge$ 1	$>160$			SR3-WZ-1Jc

## Background estimation and validation

The SM backgrounds can be classified into irreducible backgrounds with prompt leptons and genuine $E_{\text {T}}^{\text {miss}}$ from neutrinos, and reducible backgrounds that contain one or more “fake” or non-prompt (FNP) leptons or where experimental effects (e.g., detector mismeasurement of jets or leptons or imperfect removal of object double-counting) lead to significant “fake” $E_{\text {T}}^{\text {miss}}$. A summary of the background estimation techniques used in each channel is provided in Table 4. In the 2$\ell$ + 0 jets and 3$\ell$ channels only, the dominant backgrounds are estimated from MC simulation and normalized in dedicated control regions (CRs) that are included, together with the SRs, in simultaneous likelihood fits to data, as described further in Sect. 9. In addition, all channels employ validation regions (VRs) with kinematic requirements that are similar to the SRs but with smaller expected signal-to-background ratios, which are used to validate the background estimation methodology. In the 2$\ell$ + jets channel, the MC modelling of diboson processes is studied in dedicated VRs and found to accurately reproduce data.

**Table 4:** Summary of the estimation methods used in each search channel. Backgrounds denoted CR have a dedicated control region that is included in a simultaneous likelihood fit to data to extract a data-driven normalization factor that is used to scale the MC prediction. The $\gamma$ + jet template method is used in the 2$\ell$ + jets channel to provide a data-driven estimate of the *Z* + jets background. Finally, MC stands for pure Monte Carlo estimation

Channel	2$\ell$ + 0 jets	2$\ell$ + jets	3$\ell$
Background estimation summary			
Fake/non-prompt leptons	Matrix method	Matrix method	Fake-factor method
$t\bar{t} + Wt$	CR	MC	Fake-factor method
*VV*	CR	MC	CR (WZ-only)
*Z* + jets	MC	$\gamma$ + jet template	Fake-factor method
Higgs/*VVV*/top + *V*	MC	MC	MC

For the 2$\ell$ + 0 jets channel the dominant backgrounds are irreducible processes from SM diboson production (*WW*, *WZ*, and *ZZ*) and dileptonic $t\bar{t}$ and *Wt* events. MC simulation is used to predict kinematic distributions for these backgrounds, but the $t\bar{t}$ and diboson backgrounds are then normalized to data in dedicated control regions. For the diboson backgrounds, SF and DF events are treated separately and two control regions are defined. The first one (CR2-VV-SF) selects SFOS lepton pairs with an invariant mass consistent with the *Z* boson mass and has a tight requirement of $m_\mathrm {T2}$ $>130$ GeV to reduce the *Z* + jets contamination. This region is dominated by *ZZ* events, with subdominant contributions from *WZ* and *WW* events. The DF diboson control region (CR2-VV-DF) selects events with a different flavour opposite sign pair and further requires $50<m_{\mathrm{T2}}<75$ GeV. This region is dominated by *WW* events, with a subdominant contribution from *WZ* events. The $t\bar{t}$ control region (CR2-Top) uses DF events with at least one *b*-tagged jet to obtain a high-purity sample of $t\bar{t}$ events. The control region definitions are summarized in Table 5. The *Z* + jets and Higgs boson contributions are expected to be small in the 2$\ell$ + 0 jets channel and are estimated directly from MC simulation.

**Fig. 2:**
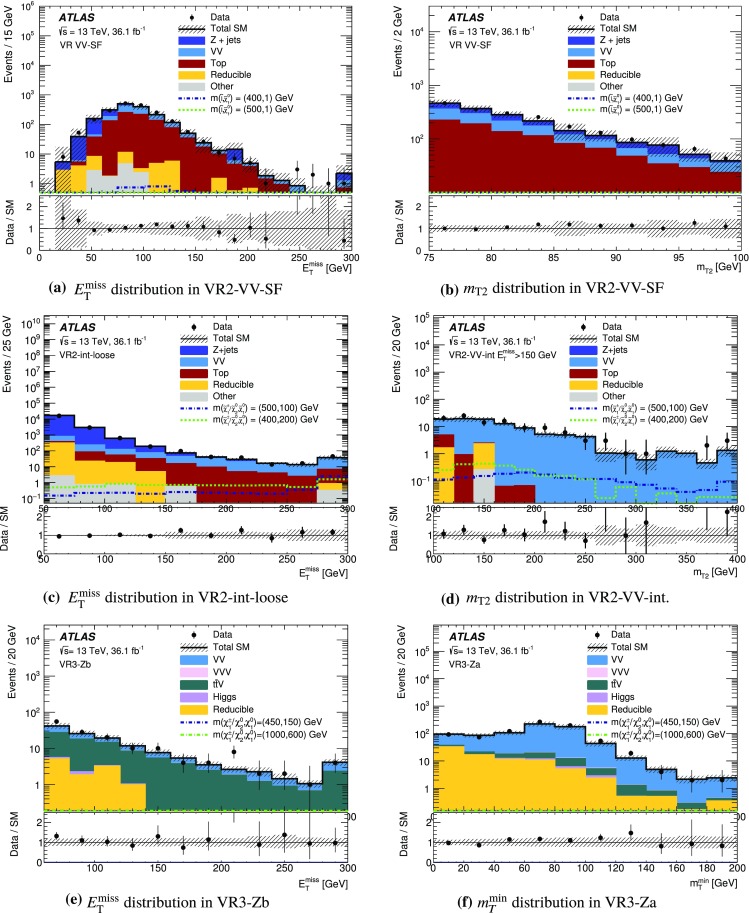
Distributions of $E_{\text {T}}^{\text {miss}}$, $m_{T}^{\mathrm {min}}$, and $m_\mathrm {T2}$ for data and the estimated SM backgrounds in the (top) 2$\ell$ + 0 jets channel, (middle) 2$\ell$ + jets channel, and (bottom) 3$\ell$ channel. Simulated signal models are overlaid for comparison. For the 2$\ell$ + 0 jets (3$\ell$) channel, the normalization factors extracted from the corresponding CRs are used to rescale the $t\bar{t}$ and *VV* (*WZ*) backgrounds. For the 2$\ell$ + 0 jets channel the “top” background includes $t\bar{t}$ and *Wt*, the “other” backgrounds include Higgs bosons, $t\bar{t}V$ and *VVV* and the “reducible” category corresponds to the data-driven matrix method estimate. For the 2$\ell$ + jets channel, the “top” background includes $t\bar{t}$, *Wt* and $t\bar{t}V$, the “other” backgrounds include Higgs bosons and *VVV*, the “reducible” category corresponds to the data-driven matrix method estimate, and the *Z* + jets contribution is evaluated with the data-driven $\gamma$ + jet template method. For the 3$\ell$ channel, the “reducible” category corresponds to the data-driven fake-factor estimate. The uncertainty band includes all systematic and statistical sources and the final bin in each histogram also contains the events in the overflow bin

The three control regions are included in a simultaneous profile likelihood fit to the observed data which provides data-driven normalization factors for these backgrounds, as described in Sect. 9. The results are propagated to the signal regions, and to dedicated VRs that are defined in Table 5. The normalization factors returned by the fit for the $t\bar{t}$, VV-DF and VV-SF backgrounds are $0.95\pm 0.03$, $1.06\pm 0.18$ and $0.96\pm 0.11$, respectively. Figure 2a, b show the $E_{\text {T}}^{\text {miss}}$ and $m_{\mathrm {T2}}$ distributions, respectively, for data and the estimated backgrounds in VR2-VV-SF with these normalization factors applied.

**Table 5:** Control region and validation region definitions for the 2$\ell$ + 0 jets channel. The DF and SF labels refer to different-flavour or same-flavour lepton pair combinations, respectively. The $p_{\text {T}}$ thresholds placed on the requirements for *b*-tagged and non-*b*-tagged jets correspond to 20 GeV and 60 GeV, respectively

Region	CR2-VV-SF	CR2-VV-DF	CR2-Top	VR2-VV-SF (DF)	VR2-Top
2$\ell$ + 0 jets control and validation region definitions					
Lepton flavour	SF	DF	DF	SF (DF)	DF
$n_{\text {non-} b \text {-tagged jets}}$	0	0	0	0	0
$n_{b \text {-tagged jets}}$	0	0	$\ge 1$	0	$\ge 1$
$|m_{\ell \ell }-m_Z|$ [GeV]	$<20$	–	–	$>20$ (–)	–
$m_{\mathrm {T2}}$ [GeV]	$>130$	50–75	75–100	75–100	$>100$

In the 2$\ell$ + jets channel, the largest background contribution is also from SM diboson production. In addition, *Z* + jets events can enter the SRs due to fake $E_{\text {T}}^{\text {miss}}$ from jet or lepton mismeasurements or genuine $E_{\text {T}}^{\text {miss}}$ from neutrinos in semileptonic decays of *b*- or *c*-hadrons. These effects are difficult to model in MC simulation, so instead $\gamma {+}\text {jets}$ events in data are used to extract the $E_{\text {T}}^{\text {miss}}$ shape in $Z{+}\text {jets}$ events, which have a similar topology and $E_{\text {T}}^{\text {miss}}$ resolution. Similar methods have been employed in searches for SUSY in events with two leptons, jets, and large $E_{\text {T}}^{\text {miss}}$ in ATLAS [90] and CMS [91, 92]. The $E_{\text {T}}^{\text {miss}}$ shape is extracted from a data control sample of $\gamma {+}\text {jets}$ events using a set of single-photon triggers and weighting each event by the trigger prescale factor. Corrections to account for differences in the $\gamma$ and *Z* boson $p_{\text {T}}$ distributions, as well as different momentum resolutions for electrons, muons and photons, are applied. Backgrounds of $W\gamma$ and $Z\gamma$ production, which contain a photon and genuine $E_{\text {T}}^{\text {miss}}$ from neutrinos, are subtracted using MC samples that are normalized to data in a $V\gamma$ control region containing a selected lepton and photon. For each SR separately, the $E_{\text {T}}^{\text {miss}}$ shape is then normalized to data in a corresponding control region with $E_{\text {T}}^{\text {miss}} <100$ GeV but all other requirements the same as in the SR. To model quantities that depend on the individual lepton momenta, an $m_{\ell \ell }$ value is assigned to each $\gamma {+}\text {jets}$ event by sampling from $m_{\ell \ell }$ distributions (parameterized as functions of boson $p_{\text {T}}$ and $E_\mathrm {T,\parallel }^{\mathrm {miss}}$, the component of $E_{\text {T}}^{\text {miss}}$ that is parallel to the boson’s transverse momentum vector) extracted from a $Z{+}\text {jets}$ MC sample. With this $m_{\ell \ell }$ value assigned to the photon, each $\gamma {+}\text {jets}$ event is boosted to the rest frame of the hypothetical *Z* boson and the photon is split into two pseudo-leptons, assuming isotropic decays in the rest frame.

To validate the method, two sets of validation regions, “tight” and “loose”, are defined for each SR. The definitions of these regions are provided in Table 6. The selections in the “tight” regions are identical to the SR selections with the exception of the dijet mass $m_{jj}$ requirement, which is replaced by the requirement ($m_{jj}<60$ GeV or $m_{jj}>100$ GeV) to suppress signal. These “tight” regions are used to verify the expectation from the $\gamma {+}\text {jets}$ method that the residual $Z{+}\text {jets}$ background after applying the SR selections is very small. The “loose” validation regions are instead defined by removing several other kinematic requirements used in the SR definition ($m_\mathrm {T2}$, all $\Delta \phi$ and $\Delta R$ quantities, and the ratios of $E_{\text {T}}^{\text {miss}}$ to *W* $p_{\text {T}}$, *Z* $p_{\text {T}}$, and $p_{\text {T}}$ of the system of ISR jets). These samples have enough $Z{+}\text {jets}$ events to perform comparisons of kinematic distributions, which validate the normalization and kinematic modelling of the $Z{+}\text {jets}$ background. The data distributions are consistent with the expected background in these validation regions, as shown in Fig. 2c for the $E_{\text {T}}^{\text {miss}}$ distribution in VR2-int-loose.

Once the signal region requirements are applied, the dominant background in the 2$\ell$ + jets channel is the diboson background. This is taken from MC simulation, but the modelling is verified in two dedicated validation regions, one for signal regions with low mass-splitting (VR2-VV-low) and one for the intermediate and high-mass signal regions (VR2-VV-int). Requiring high $E_{\text {T}}^{\text {miss}}$ and exactly one signal jet (compared to at least two jets in the SRs) suppresses the $t\bar{t}$ background and enhances the purity of diboson events containing an ISR jet, in which each boson decays leptonically. Figure 2d shows the $m_\mathrm {T2}$ distribution in VR2-VV-int for data and the expected backgrounds.

**Table 6:** Validation region definitions used for the 2$\ell$ + jets channel. Symbols and abbreviations are analogous to those in Table 2

	VR2-int(high)	VR2-low-2J(3J)	VR2-VV-int	VR2-VV-low
2$\ell$ + jets validation region definitions				
Loose selection				
$n_{\text {non-} b \text {-tagged jets}}$	$\ge 2$	2 (3–5)	1	1
$E_{\text {T}}^{\text {miss}}$ [GeV]	$>150$ ($>250$)	$>100$	$>150$	$>150$
$m_{\ell \ell }$ [GeV]	81–101	81–101 (86–96)		81–101
$m_{jj}$ [GeV]	$\notin [60,100]$	$\notin [60,100]$		
$p^{Z}_\mathrm{T}$ [GeV]	$>80$	$>60$ ($>40$)		
$p^{W}_\mathrm{T}$ [GeV]	$>100$			
$|\eta (Z)|$		$(<1.6 )$		
$p^{\mathrm{jet}3}_\mathrm{T}$ [GeV]		($>30$)		
$\Delta \phi _{(\mathbf {p}_\mathrm {T}^\mathrm {miss},\mathrm{jet})}$			$>0.4$	$>0.4$
$m_\mathrm{T2}$ [GeV]			$>100$	
$\Delta R_{(\ell \ell )}$				$<0.2$
Tight selection				
$\Delta R_{(jj)}$	$<1.5$	($<2.2$)		
$\Delta \phi _{(\mathbf {p}_\mathrm {T}^\mathrm {miss},W)}$	0.5–3.0	$>1.5$ ($<2.2$)		
$$\Delta \phi _{(\mathbf {p}_\mathrm {T}^\mathrm {miss},Z)}$$		$$ <0.8$$ $$(-)$$		
$$E_{\text {T}}^{\text {miss}}/p^{W}_\mathrm{T}$$		$$ <0.8$$ $$(-)$$		
$E_{\text {T}}^{\text {miss}}/p^{Z}_\mathrm{T}$		0.6–1.6 $(-)$		
$$E_{\text {T}}^{\text {miss}}/p^\mathrm{ISR}_\mathrm{T}$$		(0.4–0.8)		
$\Delta \phi _{(\mathbf {p}_\mathrm {T}^\mathrm {miss},\mathrm{ISR})}$		$(>2.4)$		
$\Delta \phi _{(\mathbf {p}_\mathrm {T}^\mathrm {miss},\mathrm{jet1})}$		$(>2.6)$		
$m_\mathrm{T2}$ [GeV]	$>100$			
$\Delta R_{(\ell \ell )}$	$<1.8$			

For both the 2$\ell$ + 0 jets and 2$\ell$ + jets channels, reducible backgrounds with one or two FNP leptons arise from multijet, *W* + jets and single-top-quark production events. For both analyses, the FNP lepton background is estimated from data using the matrix method (MM) [93]. This method uses two types of lepton identification criteria: “signal”, corresponding to leptons passing the full analysis selection, and “baseline”, corresponding to candidate electrons and muons as defined in Sect. 5. Probabilities for real leptons satisfying the baseline selection to also satisfy the signal selection are measured as a function of $p_{\text {T}}$ and $\eta$ in dedicated regions enriched in *Z* boson processes; similar probabilities for FNP leptons are measured in events dominated by leptons from heavy flavour decays and photon conversions. The method uses the number of observed events containing baseline–baseline, baseline–signal, signal–baseline and signal–signal lepton pairs in a given SR to extract data-driven estimates for the FNP lepton background in the CRs, VRs, and SRs for each analysis.

For the 3$\ell$ channel, the irreducible background is dominated by SM *WZ* diboson processes. As in the 2$\ell$ + 0 jets channel, the shape of this background is taken from MC simulation but normalized to data in a dedicated control region. The signal regions shown in Table 3 include a set of exclusive regions inclusive in jet multiplicity which target $\tilde{\ell }$-mediated decays, and a set of exclusive regions separated into 0-jet and $\ge 1$ jet categories which target gauge-boson-mediated decays. To reflect this, three control regions are defined in order to extract the normalization of the *WZ* background: an inclusive region (CR3-WZ-inc) and two exclusive control regions (CR3-WZ-0j and CR3-WZ-1j). The results of the background estimations are validated in a set of dedicated validation regions. This includes two validation regions that are binned in jet multiplicity (VR3-Za-0J and VR3-Za-1J), and a set of inclusive validation regions (VR3-Za, VR3-Zb, VR3-offZa and VR3-offZb) targeting different regions of phase space considered in the analysis (i.e. within and outside the *Z* boson mass window, high and low $E_{\text {T}}^{\text {miss}}$, and vetoing events with a trilepton invariant mass within the *Z* boson mass window). The definitions of the control and validation regions used in the 3$\ell$ analysis are shown in Table 7. The normalization factors extracted from the fit for inclusive *WZ* events, *WZ* events with zero jets, and *WZ* events with at least one jet are $0.97\pm 0.06$, $1.08\pm 0.06$ and $0.94\pm 0.07$, respectively. Other small background sources such as *VVV*, *tV* and Higgs boson production processes contributing to the irreducible background are taken from MC simulation.

**Table 7:** Control and validation region definitions used in the 3$\ell$ channel. The $m_{\mathrm {SFOS}}$ quantity is the mass of the same-flavour opposite-sign lepton pair and $m_{{\ell \ell \ell }}$ is the trilepton invariant mass. Other symbols and abbreviations are analogous to those in Table 3

	$p^{\ell _{3}}_\mathrm{T}$ [GeV]	$m_{\mathrm {\ell \ell \ell }}$ [GeV]	$m_{\mathrm {SFOS}}$ [GeV]	$E_{\text {T}}^{\text {miss}}$ [GeV]	$m^\mathrm {min}_\mathrm {T}$ [GeV]	$n_{\text {non-} b \text {-tagged~jets}}$	$n_{ b \text {-tagged jets}}$
3$\ell$ control and validation region definitions							
CR3-WZ-inc	$>20$	–	81.2–101.2	$>120$	$<110$	–	0
CR3-WZ-0j	$>20$	–	81.2–101.2	$>60$	$<110$	0	0
CR3-WZ-1j	$>20$	–	81.2–101.2	$>120$	$<110$	$>0$	0
VR3-Za	$>30$	$\notin [81.2,101.2]$	81.2–101.2	40–60	–	–	–
VR3-Zb	$>30$	$\notin [81.2,101.2]$	81.2–101.2	>60	–	–	$>0$
VR3-offZa	$>30$	$\notin [81.2,101.2]$	$\notin [81.2,101.2]$	40–60	–	–	–
VR3-offZb	$>20$	$\notin [81.2,101.2]$	$\notin [81.2,101.2]$	> 40	–	–	$>0$
VR3-Za-0J	$>20$	$\notin [81.2,101.2]$	81.2–101.2	40–60	–	0	0
VR3-Za-1J	$>20$	$\notin [81.2,101.2]$	81.2–101.2	40–60	–	$>0$	0

In addition to processes contributing to the reducible backgrounds in the 2$\ell$ channels, the reducible backgrounds in the 3$\ell$ channel also include $Z+$jets, $t\bar{t}$, *WW* and in general any physics process leading to less than three prompt and isolated leptons. The reducible backgrounds in the 3$\ell$ channel are estimated using a data-driven fake-factor (FF) method [94]. This method uses two sets of lepton identification criteria: the tight, or “ID”, criteria corresponding to the signal lepton selection used in the analysis and the orthogonal loose, or “anti-ID”, criteria which are designed to yield an enrichment in FNP leptons. In particular, for the anti-ID leptons the isolation and identification requirements applied to signal leptons are reversed. The *Z* + jets background events in the signal, control and validation regions are estimated using lepton $p_{\text {T}}$-dependent fake factors, defined as the ratio of the numbers of ID to anti-ID leptons in an FNP-dominated region. These fake factors are then applied to events passing selection requirements identical to those in the signal, control or validation region in question but where one of the ID leptons is replaced by an anti-ID lepton. The “top-like” contamination, which includes $t\bar{t}$, *Wt*, and *WW*, is subtracted from these anti-ID regions along with contributions from any remaining MC processes, to avoid double-counting. The top-like reducible background contributions are then estimated differently: data-to-MC scale factors derived with DF opposite-sign events are applied to simulated SF events. Figure 2e, f show the $E_{\text {T}}^{\text {miss}}$ distribution in VR3-Zb and the $m_{T}^{\mathrm {min}}$ distribution in VR3-Za, respectively.

## Systematic uncertainties

Several sources of experimental and theoretical systematic uncertainty are considered in the SM background estimates and signal predictions. These uncertainties are included in the profile likelihood fit described in Sect. 9. The primary sources of systematic uncertainty are related to the jet energy scale (JES) and resolution (JER), theory uncertainties in the MC modelling, the reweighting procedure applied to simulation to match the distribution of the number of reconstructed vertices observed in data, the systematic uncertainty considered in the non-prompt background estimation and the theoretical cross-section uncertainties. The statistical uncertainty of the simulated event samples is taken into account as well. The effects of these uncertainties were evaluated for all signal samples and background processes. In the 2$\ell$ + 0jets and 3$\ell$ channels the normalizations of the MC predictions for the dominant background processes are extracted in dedicated control regions and the systematic uncertainties thus only affect the extrapolation to the signal regions in these cases.

The JES and JER uncertainties are derived as a function of jet $p_{\text {T}}$ and $\eta$, as well as of the pile-up conditions and the jet flavour composition of the selected jet sample. They are determined using a combination of data and simulation, through measurements of the jet response balance in dijet, *Z* + jets and $\gamma +$jets events [79, 80].

The systematic uncertainties related to the $E_{\text {T}}^{\text {miss}}$ modelling in the simulation are estimated by propagating the uncertainties in the energy or momentum scale of each of the physics objects, as well as the uncertainties in the soft term’s resolution and scale [95].

The remaining detector-related systematic uncertainties, such as those in the lepton reconstruction efficiency, energy scale and energy resolution, in the *b*-tagging efficiency and in the modelling of the trigger [73, 75], are included but were found to be negligible in all channels.

The uncertainties coming from the modelling of diboson events in MC simulation are estimated by varying the renormalization, factorization and merging scales used to generate the samples, and the PDFs. In the 2$\ell$ + 0 jets channel the impact of these uncertainties in the modelling of *Z* + jets events is also considered, as well as uncertainties in the modelling of $t\bar{t}$ events due to parton shower simulation (by comparing samples generated with Powheg + Pythia to Powheg + Herwig++  [61]), ISR/FSR modelling (by comparing the predictions from an event sample generated by Powheg + Pythia with those from two samples where the radiation settings are varied), and the PDF set.

In the 2$\ell$ + jets channel, uncertainties in the data-driven $Z{+}\text {jets}$ estimate are calculated following the methodology used in Ref. [90]. An additional uncertainty is based on the difference between the expected background yield from the nominal method and a second method implemented as a cross-check, which extracts the dijet mass shape from data validation regions, normalizes the shape to the sideband regions of the SRs, and extrapolates the background into the *W* mass region.

For the matrix-method and fake-factor estimates of the FNP background, systematic uncertainties are assigned to account for differences in FNP lepton composition between the SR and the CR used to derive the fake rates and fake factors. An additional uncertainty is assigned to the MC subtraction of prompt leptons from this CR.

The exclusive SRs in the 2$\ell$ + 0 jets and 3$\ell$ channels are dominated by statistical uncertainties in the background estimates (which range from 10 to 70% in the higher mass regions in the 2$\ell$ + 0 jets channel and from 5 to 30% in the 3$\ell$ channel). The largest systematic uncertainties are those related to diboson modelling, the JES and JER uncertainties and those associated with the $E_{\text {T}}^{\text {miss}}$ modelling. In the 2$\ell$ + jets channel the dominant uncertainties are those associated with the data-driven estimate of the $Z{+}\text {jets}$ background, which range from approximately 45 to 75%.

## Results

The HistFitter framework [96] is used for the statistical interpretation of the results, with the CRs (for the 2$\ell$ + 0 jets and 3$\ell$ channels) and SRs both participating in a simultaneous likelihood fit. The likelihood is built as the product of a Poisson probability density function describing the observed number of events in each CR/SR and Gaussian distributions that constrain the nuisance parameters associated with the systematic uncertainties and whose widths correspond to the sizes of these uncertainties; Poisson distributions are used instead for MC statistical uncertainties. Correlations of a given nuisance parameter among the different background sources and the signal are taken into account when relevant.

**Fig. 3:**
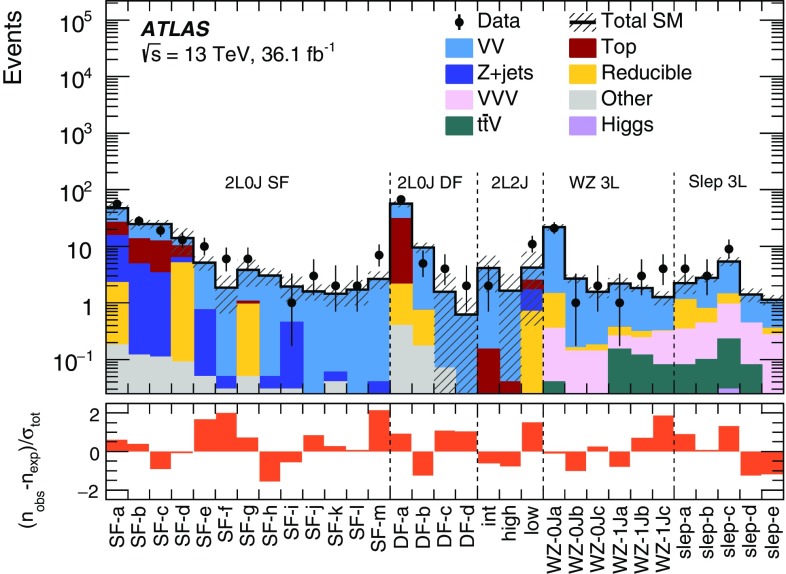
The observed and expected SM background yields in the signal regions considered in the 2$\ell$ + 0 jets, 2$\ell$ + jets and 3$\ell$ channels. The statistical uncertainties in the background prediction are included in the uncertainty band, together with the experimental and theoretical uncertainties. The bottom plot shows the difference in standard deviations between the observed and expected yields. Here $n_{\mathrm {obs}}$ and $n_{\mathrm {exp}}$ are the observed data and expected background yields, respectively, $\sigma _{\mathrm {tot}}=\sqrt{n_{\mathrm {bkg}}+\sigma ^{2}_{\mathrm {bkg}}}$, and $\sigma _{\mathrm {exp}}$ is the total background uncertainty

In the 2$\ell$ + 0 jets and 3$\ell$ channels, a background-only fit which uses data in the CRs is performed to constrain the nuisance parameters of the likelihood function (these include the normalization factors for dominant backgrounds and the parameters associated with the systematic uncertainties). In all channels the background estimates are also used to evaluate how well the expected and observed numbers of events agree in the validation regions, and good agreement is found. In the 2$\ell$ + 0 jets, 2$\ell$ + jets, and 3$\ell$ channels, the number of considered VRs is 3, 8, and 6, respectively, and the most significant deviations observed are 0.4$\sigma$, 1.4$\sigma$, and 0.8$\sigma$, respectively. The precision of the expected background yields in the VRs is significantly better than in the corresponding SRs and the dominant sources of systematic uncertainty in the VRs and corresponding SRs are similar. For the 2$\ell$ + 0 jets channel, the results for the exclusive signal regions are shown in Tables 8, 9 and 10 for SR2-SF-a to SR2-SF-g, SR2-SF-h to SR2-SF-m and SR2-DF-a to SR2-DF-d, respectively. The results for the 2$\ell$ + 0 jets inclusive signal regions are shown in Table 11, while Table 12 summarizes the expected SM background and observed events in the 2$\ell$ + jets SRs. For the 3$\ell$ channel, the results are shown in Table 13 for SR3-WZ-0Ja to SR3-WZ-0Jc and SR3-WZ-1Ja to SR3-WZ-1Jc (which target gauge-boson-mediated decays) and Table 14 for SR3-slep-a to SR3-slep-e. A summary of the observed and expected yields in all of the signal regions considered in this paper is provided in Fig. 3. No significant excess above the SM expectation is observed in any SR.

**Table 8:** Background-only fit results for SR2-SF-a to SR2-SF-g in the 2$\ell$ + 0 jets channel. All systematic and statistical uncertainties are included in the fit. The “other” backgrounds include all processes producing a Higgs boson, *VVV* or $t\bar{t}V$. A “–” symbol indicates that the background contribution is negligible

SR2-	SF-a	SF-b	SF-c	SF-d	SF-e	SF-f	SF-g
Observed	56	28	19	13	10	6	6
Total SM	$47 \pm 12$	$25 \pm 5\;\,\,$	$25 \pm 4\;\,\,$	$14 \pm 7\;\,\,$	$5.2 \pm 1.4$	$1.9 \pm 1.2$	$3.8 \pm 1.9$
$t\bar{t}$	$10 \pm 4\;\,$	$7.4 \pm 3.5$	$7.3 \pm 3.0$	$2.7 \pm 1.7$	–	–	$0.11_{-0.11}^{+0.21}\,\,$
*Wt*	$\,1.0 \pm 1.0$	$1.3 \pm 0.7$	$1.6 \pm 0.6$	$1.1 \pm 1.1$	–	–	–
*VV*	$21 \pm 4\;\,$	$11.3 \pm 2.9\;\,$	$12.6 \pm 2.4\;\,$	$3.9 \pm 2.4$	$4.4 \pm 1.3$	$1.8 \pm 1.2$	$2.8 \pm 1.6$
FNP	$2.1_{-2.1}^{+2.9}\,$	$0.0^{+0.4}_{-0.0}$	$0.0^{+0.5}_{-0.0}$	$5 \pm 4$	$0.0^{+0.1}_{-0.0}$	$0.00^{+0.01}_{-0.00}$	$0.9 \pm 0.4$
*Z* + jets	$13 \pm 9\;\,$	$4.7 \pm 2.6$	$3.3 \pm 3.2$	$1.2_{-1.2}^{+1.7}\,$	$0.7 \pm 0.6$	$0.02_{-0.02}^{+0.21}\,\,$	–
Other	$\,0.18 \pm 0.08$	$0.12 \pm 0.05$	$0.11 \pm 0.04$	$0.09 \pm 0.05$	$0.05 \pm 0.03$	$0.03 \pm 0.01$	$0.05 \pm 0.02$

**Table 9:** Background-only fit results for SR2-SF-h to SR2-SF-m in the 2$\ell$ + 0 jets channel. All systematic and statistical uncertainties are included in the fit. The “other” backgrounds include all processes producing a Higgs boson, *VVV* and $t\bar{t}V$. A “–” symbol indicates that the background contribution is negligible

SR2-	SF-h	SF-i	SF-j	SF-k	SF-l	SF-m
Observed	0	1	3	2	2	7
Total SM	$3.1 \pm 1.0$	$1.9 \pm 0.9$	$1.6 \pm 0.5$	$1.5 \pm 0.6$	$1.8 \pm 0.8$	$2.6 \pm 0.9$
$t\bar{t}$	–	–	–	–	–	–
*Wt*	–	–	–	–	–	–
*VV*	$3.0 \pm 1.0$	$1.5 \pm 0.8$	$1.6 \pm 0.5$	$1.4 \pm 0.6$	$1.7 \pm 0.8$	$2.6 \pm 0.9$
FNP	$0.00^{+0.02}_{-0.00}$	$0.0^{+0.1}_{-0.0}$	$0.00^{+0.01}_{-0.00}$	$0.00^{+0.01}_{-0.00}$	$0.00^{+0.02}_{-0.00}$	$0.00^{+0.01}_{-0.00}$
*Z* + jets	$0.02_{-0.02}^{+0.11}\,\,$	$0.42 \pm 0.20$	–	$0.02_{-0.02}^{+0.20}\,\,$	–	$0.02_{-0.02}^{+0.06}\,\,$
Other	$0.03 \pm 0.01$	$0.03 \pm 0.02$	–	$0.04 \pm 0.02$	$0.02 \pm 0.01$	$0.02 \pm 0.02$

**Table 10:** Background-only fit results for SR2-DF-a to SR2-DF-d in the 2$\ell$ + 0 jets channel. All systematic and statistical uncertainties are included in the fit. The “other” backgrounds include all processes producing a Higgs boson, *VVV* or $t\bar{t}V$. A “–” symbol indicates that the background contribution is negligible

SR2-	DF-a	DF-b	DF-c	DF-d
Observed	67	5	4	2
Total SM	$57 \pm 7\;\,\,$	$9.6 \pm 1.9$	$1.5_{-1.5}^{+1.7}\,\,$	$0.6 \pm 0.6$
$t\bar{t}$	$24 \pm 8\;\,$	–	–	–
*Wt*	$4.5 \pm 1.0$	–	–	–
*VV*	$26 \pm 6\;\,$	$8.8 \pm 1.8$	$1.5_{-1.5}^{+1.7}\,\,$	$0.6 \pm 0.6$
FNP	$1.75 \pm 0.18$	$0.57 \pm 0.23$	$0.00^{+0.01}_{-0.00}$	$0.00^{+0.01}_{-0.00}$
*Z* + jets	–	–	–	–
Other	$0.40 \pm 0.09$	$0.17 \pm 0.07$	$0.07 \pm 0.07$	$0.02 \pm 0.02$

**Table 11:** Background-only fit results for the inclusive signal regions in the 2$\ell$ + 0 jets channel. All systematic and statistical uncertainties are included in the fit. The “other” backgrounds include all processes producing a Higgs boson, *VVV* and $t\bar{t}V$. A “–” symbol indicates that the background contribution is negligible

SR2-	SF-loose	SF-tight	DF-100	DF-150	DF-200	DF-300
Observed	153	9	78	11	6	2
Total SM	$133 \pm 22\;\,\,$	$9.8 \pm 2.9$	$68 \pm 7\;\,\,$	$11.5 \pm 3.1\;\,\,$	$2.1 \pm 1.9$	$0.6 \pm 0.6$
$t\bar{t}$	$27 \pm 11$	–	$24 \pm 8\;\,$	–	–	–
*Wt*	$5.0 \pm 2.2$	–	$4.5 \pm 1.0$	–	–	–
*VV*	$70 \pm 11$	$9.6 \pm 3.0$	$37 \pm 8\;\,$	$10.8 \pm 3.0\;\,$	$2.0 \pm 1.9$	$0.6 \pm 0.6$
FNP	$6 \pm 4$	$0.0 \pm 0.0$	$2.17 \pm 0.29$	$0.42 \pm 0.23$	$0.00^{+0.01}_{-0.00}$	$0.00^{+0.01}_{-0.00}$
*Z* + jets	$23 \pm 14$	$0.09_{-0.09}^{+0.34}\,\,$	–	–	–	–
Other	$0.79 \pm 0.23$	$0.09 \pm 0.01$	$0.67 \pm 0.16$	$0.26 \pm 0.08$	$0.09 \pm 0.07$	$0.02 \pm 0.02$

**Table 12:** SM background results in the 2$\ell$ + jets SRs. All systematic and statistical uncertainties are included. The “top” background includes all processes producing one or more top quarks and the “other” backgrounds include all processes producing a Higgs boson or *VVV*. A “–” symbol indicates that the background contribution is negligible

SR2-	int	high	low (combined)
Observed	2	0	11
Total SM	$4.1^{+2.6}_{-1.8}\,\,$	$1.6^{+1.6}_{-1.1} \,\,$	$4.2^{+3.4}_{-1.6}\,\,$
*VV*	$4.0 \pm 1.8$	$1.6 \pm 1.1$	$1.7 \pm 1.0$
Top	$0.15 \pm 0.11$	$0.04 \pm 0.03$	$0.8 \pm 0.4$
FNP	$0.0^{+0.2}_{-0.0}\,$	$0.0^{+0.1}_{-0.0}\,$	$0.7^{+1.8}_{-0.7}\,$
$Z{+}\text {jets}$	$0.0^{+1.8}_{-0.0}\,$	$0.0^{+1.2}_{-0.0}\,$	$1.0^{+2.7}_{-1.0}\,$
Other	–	–	–

**Table 13:** Background-only fits for SR3-WZ-0Ja to SR3-WZ-0Jc and SR3-WZ-1Ja to SR3-WZ-1Jc in the 3$\ell$ channel. All systematic and statistical uncertainties are included in the fit

SR3-	WZ-0Ja	WZ-0Jb	WZ-0Jc	WZ-1Ja	WZ-1Jb	WZ-1Jc
Observed	21	1	2	1	3	4
Total SM	$21.7 \pm 2.9\;\,$	$2.7 \pm 0.5$	$1.56 \pm 0.33$	$2.2 \pm 0.5$	$1.82 \pm 0.26$	$1.26 \pm 0.34$
*WZ*	$19.5 \pm 2.9\;\,$	$2.5 \pm 0.5$	$1.33 \pm 0.31$	$1.8 \pm 0.5$	$1.49 \pm 0.22$	$0.92 \pm 0.28$
*ZZ*	$0.81 \pm 0.23$	$0.06 \pm 0.03$	$0.05 \pm 0.01$	$0.05 \pm 0.02$	$0.02 \pm 0.01$	–
*VVV*	$0.31 \pm 0.07$	$0.13 \pm 0.04$	$0.13 \pm 0.03$	$0.11 \pm 0.02$	$0.12 \pm 0.03$	$0.23 \pm 0.05$
$t\bar{t}V$	$0.04 \pm 0.02$	$0.01 \pm 0.01$	$0.01 \pm 0.01$	$0.14 \pm 0.04$	$0.12 \pm 0.02$	$0.08 \pm 0.02$
Higgs	–	–	–	$0.01 \pm 0.00$	–	–
FNP	$1.1 \pm 0.5$	$0.02 \pm 0.01$	$0.04 \pm 0.02$	$0.11 \pm 0.06$	$0.07 \pm 0.04$	$0.01 \pm 0.00$

**Table 14:** Background-only fits for SR3-slep-a to SR3-slep-e in the 3$\ell$ channel. All systematic and statistical uncertainties are included in the fit

SR3-	slep-a	slep-b	slep-c	slep-d	slep-e
Observed	4	3	9	0	0
Total SM	$2.2 \pm 0.8$	$2.8 \pm 0.4$	$5.4 \pm 0.9$	$1.4 \pm 0.4$	$1.14 \pm 0.23$
*WZ*	$1.1 \pm 0.4$	$1.98 \pm 0.31$	$3.9 \pm 0.7$	$0.91 \pm 0.26$	$0.76 \pm 0.17$
*ZZ*	$0.02 \pm 0.01$	$0.01 \pm 0.01$	$0.13 \pm 0.03$	$0.06 \pm 0.02$	$0.03 \pm 0.01$
*VVV*	$0.26 \pm 0.08$	$0.34 \pm 0.05$	$0.72 \pm 0.12$	$0.36 \pm 0.10$	$0.25 \pm 0.05$
$t\bar{t}V$	$0.07 \pm 0.03$	$0.09 \pm 0.02$	$0.20 \pm 0.04$	$0.07 \pm 0.02$	$0.02 \pm 0.01$
Higgs	$0.01 \pm 0.00$	$0.01 \pm 0.01$	$0.03 \pm 0.02$	$0.01 \pm 0.00$	–
FNP	$0.80 \pm 0.46$	$0.36 \pm 0.18$	$0.48 \pm 0.25$	–	$0.08 \pm 0.04$

**Fig. 4:**
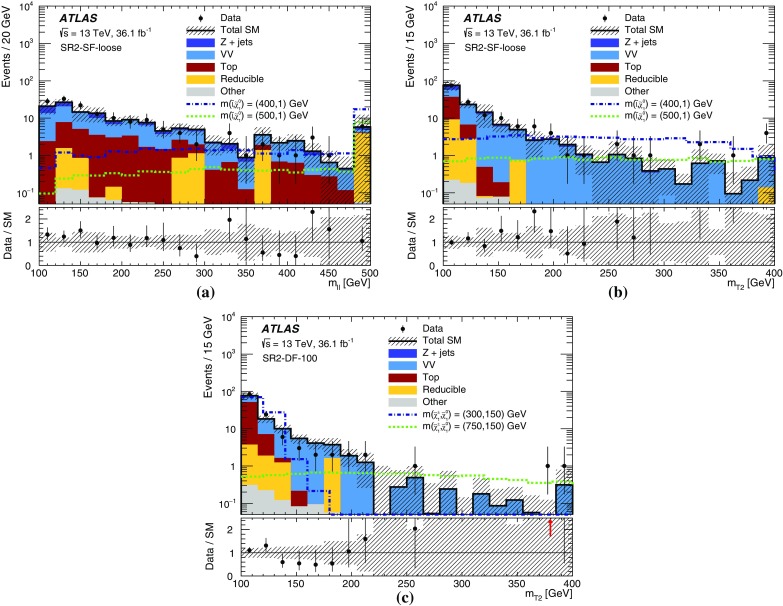
The **a** $m_{\ell \ell }$ and **b** $m_\mathrm {T2}$ distributions for data and the estimated SM backgrounds in the 2$\ell$ + 0 jets channel for SR2-SF-loose and **c** the $m_\mathrm {T2}$ distribution for the SR2-DF-100 selection. The normalization factors extracted from the corresponding CRs are used to rescale the $t\bar{t}$ and *VV* contributions. The “top” background includes $t\bar{t}$ and *Wt*, and the “other” backgrounds include Higgs bosons, $t\bar{t}V$ and *VVV*. The “reducible” category corresponds to the data-driven matrix method’s estimate. The uncertainty bands include all systematic and statistical contributions. Simulated signal models for sleptons (**a**, **b**) or charginos (**c**) pair production are overlayed for comparison. The final bin in each histogram also contains the events in the overflow bin. The vertical red arrows indicate bins where the ratio of data to SM background, minus the uncertainty on this quantity, is larger than the *y*-axis maximum

**Fig. 5:**
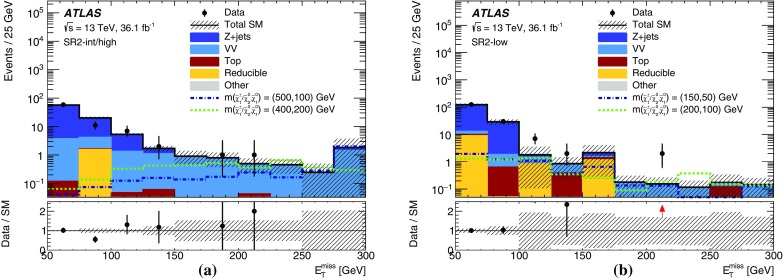
Distributions of $E_{\text {T}}^{\text {miss}}$ for data and the expected SM backgrounds in the 2$\ell$ + jets channel for **a** SR2-int/high and **b** SR2-low, without the final $E_{\text {T}}^{\text {miss}}$ requirement applied. The “top” background includes $t\bar{t}$ , *Wt* and $t\bar{t}V$, and the “other” backgrounds include Higgs bosons and *VVV*. The *Z* + jets contribution is evaluated using the data-driven $\gamma$ + jet template method and the “reducible” category corresponds to the data-driven matrix method’s estimate. The uncertainty bands include all systematic and statistical contributions. Simulated signal models for charginos/neutralinos production are overlayed for comparison. The final bin in each histogram also contains the events in the overflow bin. The vertical red arrows indicate bins where the ratio of data to SM background, minus the uncertainty on this quantity, is larger than the *y*-axis maximum

**Fig. 6:**
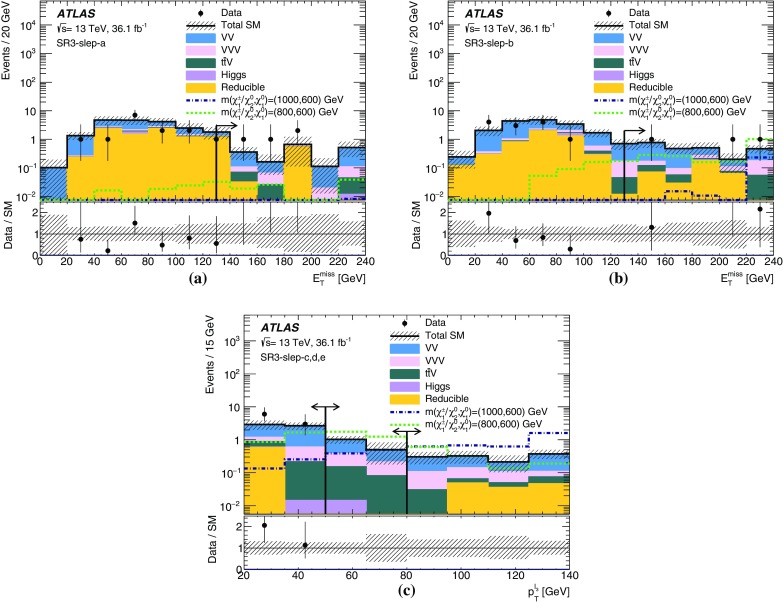
Distributions of $E_{\text {T}}^{\text {miss}}$ for data and the estimated SM backgrounds in the 3$\ell$ channel for **a** SR3-slep-a and **b** SR3-slep-b and **c** distributions of the third leading lepton $p_{\text {T}}$ in SR3-slep-c,d,e. The normalization factors extracted from the corresponding CRs are used to rescale the *WZ* background. The “reducible” category corresponds to the data-driven fake-factor estimate. The uncertainty bands include all systematic and statistical contributions. Simulated signal models for charginos/neutralinos production are overlayed for comparison. The final bin in each histogram also contains the events in the overflow bin

**Fig. 7:**
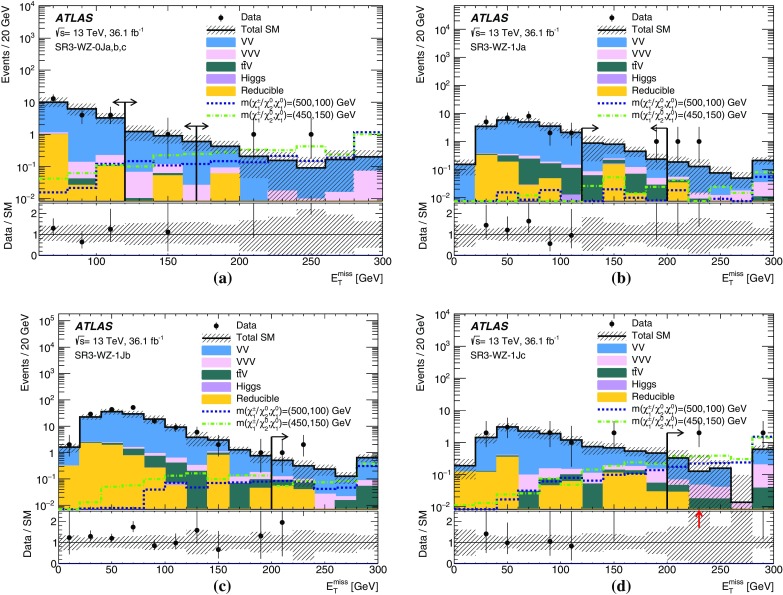
Distributions of $E_{\text {T}}^{\text {miss}}$ for data and the estimated SM backgrounds in the 3$\ell$ channel for **a** SR3-WZ-0Ja,b,c, **b** SR3-WZ-1Ja, **c** SR3-WZ-1Jb and **d** SR3-WZ-1Jc. The normalization factors extracted from the corresponding CRs are used to rescale the 0-jet and $\ge$ 1-jet *WZ* background components. The “reducible” category corresponds to the data-driven fake-factor estimate. The uncertainty bands include all systematic and statistical contributions. Simulated signal models for charginos/neutralinos production are overlayed for comparison. The final bin in each histogram also contains the events in the overflow bin. The vertical red arrows indicate bins where the ratio of data to SM background, minus the uncertainty on this quantity, is larger than the *y*-axis maximum

Figure 4 shows a selection of kinematic distributions for data and the estimated SM backgrounds with their associated statistical and systematic uncertainties for the loosest inclusive SRs in the 2$\ell$ + 0 jets channel: SR2-SF-loose and SR2-DF-100. The normalization factors extracted from the corresponding CRs are propagated to the *VV* and $t\bar{t}$ contributions. Figure 5 shows the $E_{\text {T}}^{\text {miss}}$ distribution in SR2-int and SR2-high, which differ only in the $E_{\text {T}}^{\text {miss}}$ requirement, and in SR2-low of the 2$\ell$ + jets channel. In the 3$\ell$ channel, distributions of $E_{\text {T}}^{\text {miss}}$ and the third leading lepton $p_{\text {T}}$ are shown for the SR bins targeting $\tilde{\ell }$-mediated decays in Fig. 6 while Fig. 7 shows distributions of $E_{\text {T}}^{\text {miss}}$ in the bins targeting gauge-boson-mediated decays. Good agreement between data and expectations is observed in all distributions within the uncertainties.

**Fig. 8:**
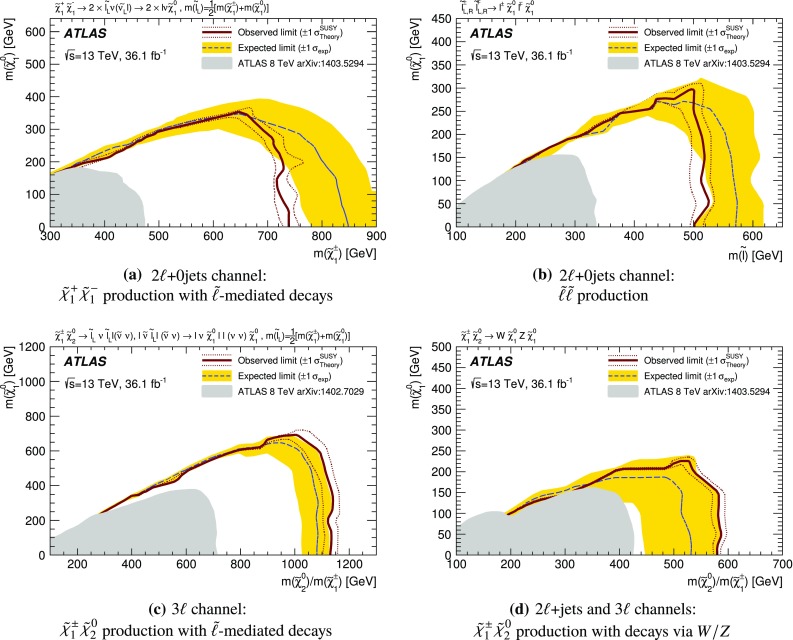
Observed and expected exclusion limits on SUSY simplified models for **a** chargino-pair production, **b** slepton-pair production, **c** chargino–neutralino production with slepton-mediated decays, and **d** chargino–neutralino production with decays via *W* / *Z* bosons. The observed (solid thick red line) and expected (thin dashed blue line) exclusion contours are indicated. The shaded band corresponds to the ±1$\sigma$ variations in the expected limit, including all uncertainties except theoretical uncertainties in the signal cross-section. The dotted lines around the observed limit illustrate the change in the observed limit as the nominal signal cross-section is scaled up and down by the theoretical uncertainty. All limits are computed at 95% confidence level. The observed limits obtained from ATLAS in Run 1 are also shown [23]

In the absence of any significant excess, two types of exclusion limits for new physics scenarios are calculated using the CL$_\mathrm {s}$ prescription [97]. First, exclusion limits are set on the masses of the charginos, neutralinos, and sleptons for the simplified models in Fig. 1, as shown in Fig. 8. Figure 8a, b show the limits in the 2$\ell$ + 0 jets channel in the models of direct chargino pair production with decays via sleptons and direct slepton pair production, respectively. Limits are calculated by statistically combining the mutually orthogonal exclusive SRs. For the chargino pair model, all SF and DF bins are used and chargino masses up to 750 GeV are excluded at 95% confidence level for a massless $\displaystyle \tilde{\chi }^0_1$ neutralino. In the region with large chargino mass, the observed limit is weaker than expected because the data exceeds the expected backgrounds in SF-e, SF-f, and SF-g. For the slepton pair model, which assumes mass-degenerate $\tilde{\ell } _{L }$ and $\tilde{\ell } _{R }$ states (where $\tilde{\ell } =\tilde{e},\tilde{\mu },\tilde{\tau }$), only SF bins are used and slepton masses up to 500 GeV are excluded for a massless $\displaystyle \tilde{\chi }^0_1$ neutralino.

Figure 8c shows the limits from the 3$\ell$ channel in the model of mass-degenerate chargino–neutralino pair production with decays via sleptons, calculated using a statistical combination of the five SR3-slep regions. In this model, chargino and neutralino masses up to 1100 GeV are excluded for $\displaystyle \tilde{\chi }^0_1$ neutralino masses less than 550 GeV.

Figure 8d shows the limits from the 3$\ell$ and 2$\ell$ + jets channels in the model of mass-degenerate chargino–neutralino pair production with decays via *W* / *Z* bosons. The 3$\ell$ limits are calculated using a statistical combination of the six SR3-WZ regions. Since the SRs in the 2$\ell$ + jets channel are not mutually exclusive, the observed CL$_\mathrm {s}$ value is taken from the signal region with the best expected CL$_\mathrm {s}$ value. The 3$\ell$ and 2$\ell$ + jets channels are then combined, using the channel with the best expected CL$_\mathrm {s}$ value for each point in the model parameter space. In this model, chargino and neutralino masses up to 580 GeV are excluded for a massless $\displaystyle \tilde{\chi }^0_1$ neutralino.

Second, model-independent upper limits are set on the visible signal cross-section ($\langle \epsilon \mathrm{\sigma }\rangle _{\mathrm{obs}}^{95}$) as well as on the observed ($S^{95}_{\mathrm{obs}}$) and expected ($S^{95}_{\mathrm{exp}}$) number of events from processes beyond-the-SM in the signal regions considered in this analysis. The *p*-value and the corresponding significance for the background-only hypothesis are also evaluated. For the 2$\ell$ + 0 jets channel the inclusive signal regions defined in Table 1 are considered whereas for the 3$\ell$ channel the calculation is performed for each bin separately. All the limits are at 95% confidence level. The results can be found in Table 15.

**Table 15:** Summary of results and model-independent limits in the inclusive 2$\ell$ + 0 jets, 2$\ell$ + jets, and 3$\ell$ SRs. The observed ($N_{\mathrm{obs}}$) and expected background ($N_{\mathrm{exp}}$) yields in the signal regions are indicated. Signal model-independent upper limits at 95% confidence level on the visible signal cross-section ($\langle \epsilon \mathrm{\sigma }\rangle _{\mathrm{obs}}^{95}$), and the observed and expected upper limit on the number of BSM events ($S^{95}_{\mathrm{obs}}$ and $S^{95}_{\mathrm{exp}}$, respectively) are also shown. The ±1$\sigma$ variations of the expected limit originate from the statistical and systematic uncertainties in the background prediction. The last two columns show the *p* value and the corresponding significance for the background-only hypothesis. For SRs where the data yield is smaller than expected, the *p* value is truncated at 0.5 and the significance is set to 0

Signal channel	Region	$N_{\mathrm{obs}}$	$N_{\mathrm{exp}}$	$\langle \epsilon \mathrm{\sigma }\rangle _{\mathrm{obs}}^{95}$[fb]	$S_{\mathrm{obs}}^{95}$	$S_{\mathrm{exp}}^{95}$	*p*(*s* = 0)	*Z*
2$\ell$ + 0 jets	DF-100	78	$68 \pm 7\;\,\,$	0.88	32	${ 27}^{ +11 }_{ -8 }\;$	0.22	0.77
	DF-150	11	$11.5 \pm 3.1\;\,\,$	0.32	11.4	${ 12 }^{ +5 }_{ -4 }\;\,\,$	0.5	0
	DF-200	6	$2.1\pm 1.9$	0.33	12.0	${ 10.3 }^{ +2.9 }_{ -1.9 }\;\,\,$	0.06	1.5
	DF-300	2	$$0.6\pm 0.6$$	0.18	6.6	$$ { 5.6 }^{ +1.1 }_{ -0.9 }$$	0.10	1.3
	SF-loose	153	$$133\pm 22\;\,\,$$	2.02	73	$$ { 53 }^{ +21 }_{ -16 }$$	0.16	1.0
	SF-tight	9	$9.8\pm 2.9$	0.29	10.5	${ 12}^{ +4 }_{ -3 }\;\,\,$	0.5	0
2$$\ell $$ + jets	SR2-int	2	$$4.1^{+2.6}_{-1.8}\;\,$$	0.13	4.5	$$ { 5.6 }^{ +2.2 }_{ -1.4 }$$	0.5	0
	SR2-high	0	$$1.6^{+1.6}_{-1.1}\;\,$$	0.09	3.1	$$ { 3.1 }^{ +1.4 }_{ -0.1 }$$	0.5	0
	SR2-low	11	$4.2^{+3.4}_{-1.6}\;\,$	0.43	15.7	${ 12 }^{ +4 }_{ -2 }\;\,\,$	0.06	1.6
3$\ell$	WZ-0Ja	21	$21.7 \pm 2.9\;\,$	0.35	12.8	${ 14 }^{ +3 }_{ -5 }\;\,\,$	0.5	0
	WZ-0Jb	1	$$2.7 \pm 0.5$$	0.10	3.7	$$ { 4.6 }^{ +2.1 }_{ -0.9 }$$	0.5	0
	WZ-0Jc	2	$$1.6 \pm 0.3$$	0.13	4.8	$$ { 4.1 }^{ +1.7 }_{ -0.7 }$$	0.28	0.57
	WZ-1Ja	1	$$2.2 \pm 0.5$$	0.09	3.2	$$ { 4.5 }^{ +1.6 }_{ -1.3 }$$	0.5	0
	WZ-1Jb	3	$$1.8 \pm 0.3$$	0.16	5.6	$$ { 4.3 }^{ +1.7 }_{ -0.9 }$$	0.18	0.91
	WZ-1Jc	4	$$1.3 \pm 0.3$$	0.20	7.2	$$ { 4.2 }^{ +1.7 }_{ -0.4 }$$	0.03	1.8
	slep-a	4	$$2.2 \pm 0.8$$	0.19	6.8	$$ { 4.7 }^{ +2.3 }_{ -0.5 }$$	0.23	0.72
	slep-b	3	$$2.8 \pm 0.4$$	0.14	5.2	$$ { 5.1 }^{ +1.9 }_{ -1.2 }$$	0.47	0.08
	slep-c	9	$$5.4 \pm 0.9$$	0.29	10.5	$$ { 6.8 }^{ +2.9 }_{ -1.3 }$$	0.09	1.4
	slep-d	0	$$1.4 \pm 0.4$$	0.08	3.0	$$ { 3.6 }^{ +1.2 }_{ -0.6 }$$	0.5	0
	slep-e	0	$$1.1 \pm 0.2$$	0.09	3.3	$$ { 3.6 }^{ +1.3 }_{ -0.5 }$$	0.5	0

## Conclusion

Searches for the electroweak production of neutralinos, charginos and sleptons decaying into final states with exactly two or three electrons or muons and missing transverse momentum are performed using 36.1 fb$^{-1}$ of $\sqrt{s}=13$ TeV proton–proton collisions recorded by the ATLAS detector at the Large Hadron Collider. Three different search channels are considered. The 2$\ell$ + 0 jets channel targets direct $\displaystyle \tilde{\chi }^+_1 \displaystyle \tilde{\chi }^-_1$ production where each $\displaystyle \tilde{\chi }^\pm _1$ decays via an intermediate $\tilde{\ell }$, and direct $\tilde{\ell } \tilde{\ell }$ production. The 2$\ell$ + jets channel targets associated $\displaystyle \tilde{\chi }^\pm _1 \displaystyle \tilde{\chi }^0_2$ production where each sparticle decays via an SM gauge boson giving a final state with two leptons consistent with a *Z* boson and two jets consistent with a *W* boson. Finally, the 3$\ell$ channel targets associated $\displaystyle \tilde{\chi }^\pm _1 \displaystyle \tilde{\chi }^0_2$ production with decays via either intermediate $\tilde{\ell }$ or gauge bosons.

No significant excess above the SM expectation is observed in any of the signal regions considered across the three channels, and the results are used to calculate exclusion limits at 95% confidence level in several simplified model scenarios. For associated $\displaystyle \tilde{\chi }^\pm _1 \displaystyle \tilde{\chi }^0_2$ production with $\tilde{\ell }$-mediated decays, masses up to 1100 GeV are excluded for $\displaystyle \tilde{\chi }^0_1$ neutralino masses less than 550 GeV. Both the 2$\ell$ + jets and 3$\ell$ channels place exclusion limits on associated $\displaystyle \tilde{\chi }^\pm _1 \displaystyle \tilde{\chi }^0_2$ production with gauge-boson-mediated decays. For a massless $\displaystyle \tilde{\chi }^0_1$ neutralino, $\displaystyle \tilde{\chi }^\pm _1$/$\displaystyle \tilde{\chi }^0_2$ masses up to approximately 580 GeV are excluded. In the 2$\ell$ + 0 jets channel, for direct $\displaystyle \tilde{\chi }^+_1 \displaystyle \tilde{\chi }^-_1$ production with decays via an intermediate $\tilde{\ell }$, masses up to 750 GeV are excluded for a massless $\displaystyle \tilde{\chi }^0_1$ neutralino. For $\tilde{\ell } \tilde{\ell }$ production, masses up to 500 GeV are excluded for a massless $\displaystyle \tilde{\chi }^0_1$ neutralino, assuming mass-degenerate $\tilde{\ell } _{L }$ and $\tilde{\ell } _{R }$ (where $\tilde{\ell } =\tilde{e},\tilde{\mu },\tilde{\tau }$). These results significantly improve upon previous exclusion limits based on Run 1 data.

## Footnotes

1 The SUSY partners of the Higgs field (known as higgsinos) and of the electroweak gauge fields (the bino for the U(1) gauge field and winos for the *W* fields) mix to form the mass eigenstates known as charginos and neutralinos.

2 ATLAS uses a right-handed coordinate system with its origin at the nominal interaction point (IP) in the centre of the detector and the *z*-axis along the beam direction. The *x*-axis points from the IP to the centre of the LHC ring, and the *y*-axis points upward. Cylindrical coordinates (*r*, $\phi$) are used in the transverse plane, $\phi$ being the azimuthal angle around the beam direction. The pseudorapidity is defined in terms of the polar angle $\theta$ as $\eta = -\ln \tan (\theta /2)$. Angular distance is measured in units of $\Delta R \equiv \sqrt{ (\Delta \eta )^2 + (\Delta \phi )^2 }$. The transverse momentum, $p_{\text {T}}$, and energy, $E_{\text {T}}$, are defined with respect to the beam axis (*x*–*y* plane).
